# Supplementary material for: Human endothelial cells promote a human neural stem cell type B phenotype via Notch signaling
Source: Nat Commun. 2025 May 30;16:5031. doi: 10.1038/s41467-025-60194-6 (PMC12125299; doi:10.1038/s41467-025-60194-6)
Supplement: Supplementary file 1 — Supplementary Information [file 41467_2025_60194_MOESM1_ESM.pdf]

Supplementary Information

Human endothelial cells promote a human neural stem cell type B phenotype via Notch signaling

Brenda Gutierrez*[1,2], Tzu Chia Liu[1,2], Carly Rodriguez[2], Oier Pastor-Alonso[3], Hannah Lambing[3], Mercedes F. Paredes[3], Lisa A. Flanagan*[1,2,4,5]

[1]Department of Anatomy & Neurobiology, University of California Irvine, Irvine, CA 92697, USA

[2]Sue & Bill Gross Stem Cell Research Center, University of California Irvine, Irvine, CA 92697, USA

[3]Neurology Department and Weill Institute for Neuroscience, University of California San Francisco, San Francisco, CA 94143, USA

[4]Department of Biomedical Engineering, University of California Irvine, Irvine, CA 92697, USA

[5]Department of Neurology, University of California Irvine, Irvine, CA 92697, USA

* Correspondence: lisa.flanagan@uci.edu and bgutier4@uci.edu

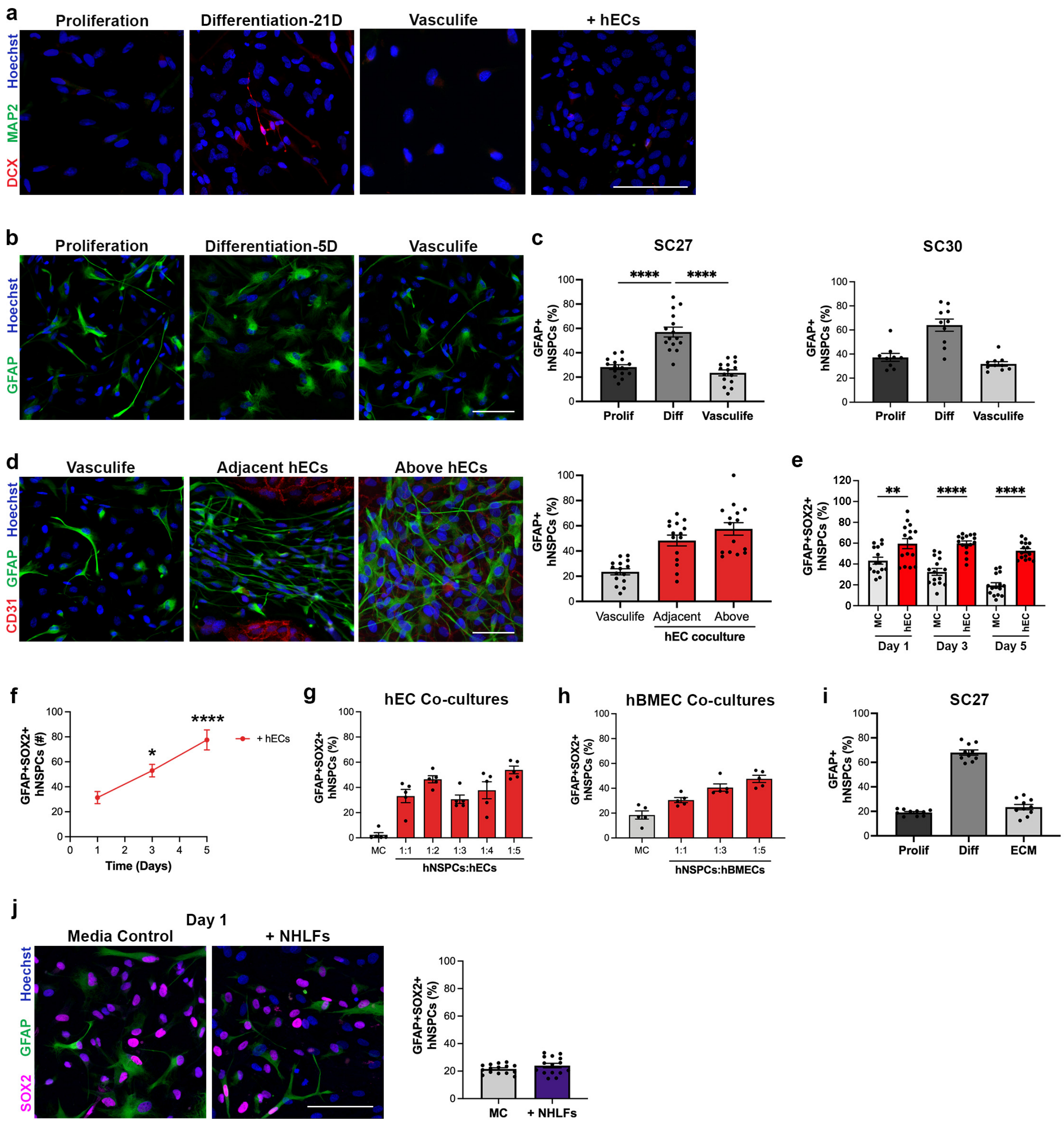

Supplementary Figure 1. Increase in the percentage of GFAP+SOX2+ hNSPCs after hEC co-culture is consistent across conditions and is specific to hECs
(a) SC27 hNSPCs in proliferation media (5 days), differentiation media (21 days), Vasculife (used for co-cultures, 5 days), and with hECs in Vasculife (5 days) were stained for neuronal markers, microtubule associated protein 2 (MAP2) and doublecortin (DCX). **(b)** SC27 hNSPCs in proliferation, differentiation, and Vasculife media for 5 days were stained for GFAP. **(c)** Percentage of GFAP+ SC27 hNSPCs was not significantly different in Vasculife compared to proliferation media (Prolif) (p=0.4977). Differentiated hNSPCs (Diff) showed higher GFAP+ percentage compared to Prolif (****p<0.0001) and Vasculife (****p<0.0001). A similar trend was seen with SC30 hNSPCs (n=2). **(d)** SC27 hNSPCs in hEC co-culture stained for GFAP and

CD31 show hNSPCs adjacent and above hECs. Vasculife data is the same as (c) (n=1 hEC co-culture). **(e)** GFAP+SOX2+ SC27 hNSPCs demonstrated a significant increase one day after co-culture (+hECs) compared to media control (MC) (**p=0.0090). The difference was more pronounced at days 3 (****p<0.0001) and 5 (****p<0.0001). **(f)** SC27 GFAP+SOX2+ hNSPCs in co-cultures (+hECs) significantly increased at days 3 (*p=0.0438) and 5 (****p<0.0001) compared to day 1. **(g)** GFAP+SOX2+ SC27 hNSPCs at different hNSPC:hEC ratios demonstrated an increase at all ratios compared to MC (n=1). Data from the 1:5 ratio is a replicate in Figure 1b and shown for comparison. **(h)** GFAP+SOX2+ SC27 hNSPCs at different hNSPC:hBMEC ratios demonstrated an increase at all ratios but a more prominent increase at 1:5 ratio (n=1). Data from the 1:5 ratio is a replicate in Figure 1d and shown for comparison. **(i)** GFAP+ SC27 hNSPCs in proliferation (Prolif), differentiation (Dif), and ECM media used in hBMEC co-cultures showed no difference in GFAP+ percentage in ECM compared to proliferation media while differentiated hNSPCs had a higher percentage of GFAP+ hNSPCs (n=2). ECM data is found in Figure 1d as 2 MC replicates. **(j)** SC27 hNSPCs in MC or with normal human lung fibroblasts (+NHLFs) for 1 day were immunostained for GFAP and SOX2. GFAP+SOX2+ percentage was not significantly different between MC and NHLF co-culture (p=0.1902). Nuclei stained with Hoechst. Scale bars, 100 μm, n=3 independent biological replicates unless otherwise specified, 5 areas quantified per sample, percentages are out of total hNSPCs, mean with SEM. Analysis for (c, f) one-way ANOVA with Tukey post-hoc test for multiple comparisons and (e, j) unpaired two-tailed Student's t-test. Source data are provided as a Source Data file.

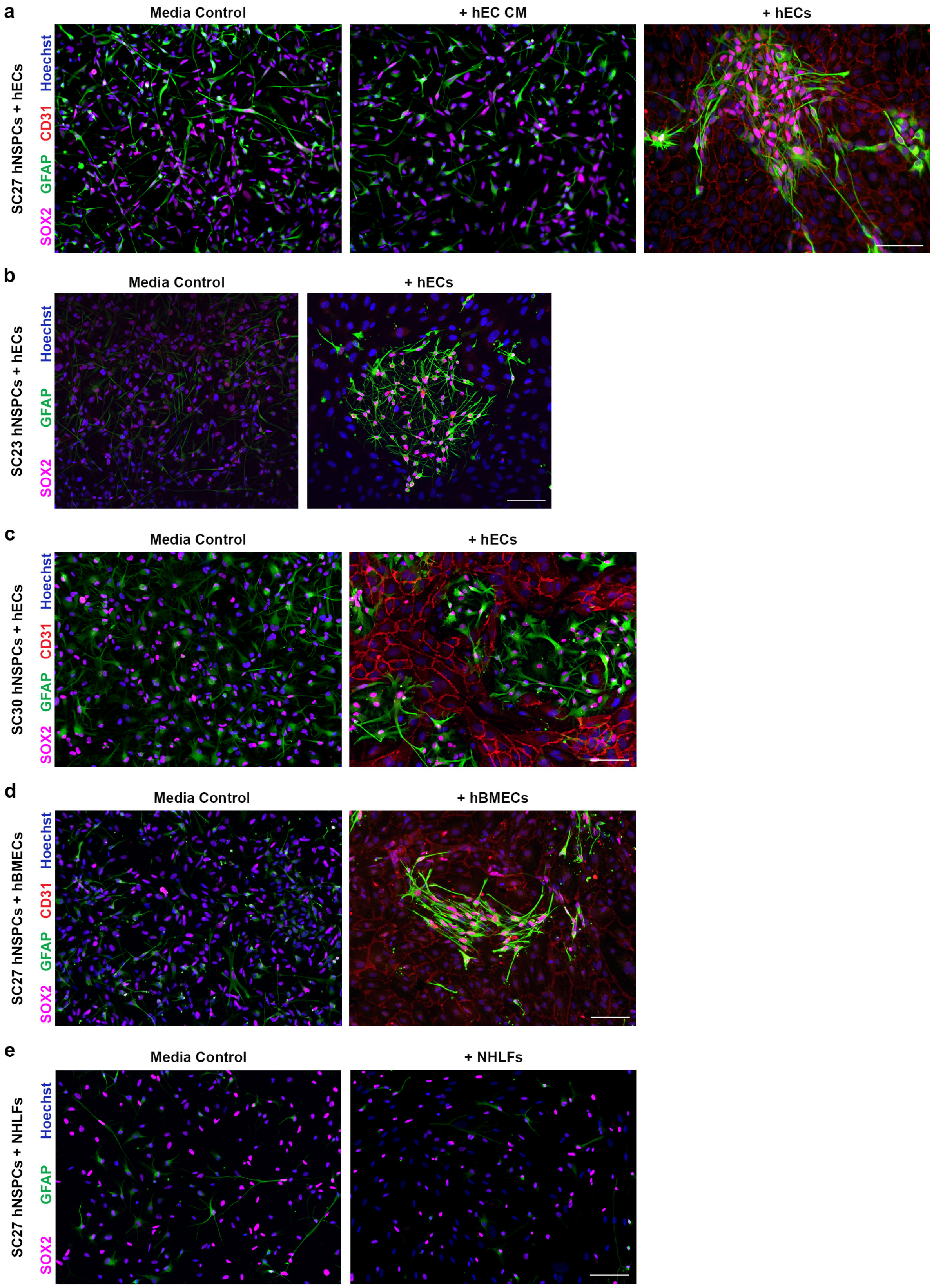

Supplementary Figure 2. hECs and hBMECs increase GFAP+SOX2+ human type B cells

but human fibroblasts do not (lower magnification images related to Figure 1)
(a) SC27 hNSPCs in media control, hEC conditioned media (hEC CM), or co-culture with hECs (+ hECs) immunostained for GFAP, SOX2 and EC marker CD31. **(b)** SC23 hNSPCs in media control or + hECs immunostained for GFAP and SOX2. **(c)** SC30 hNSPCs in media control or + hECs immunostained for GFAP, SOX2 and CD31. **(d)** SC27 hNSPCs in media control or with human brain hBMEC co-cultures (+ hBMECs) immunostained for GFAP, SOX2 and CD31. **(e)** SC27 hNSPCs in media control or NHLF co-culture (+NHLFs) immunostained for GFAP and SOX2. All images are day 5 co-cultures. Nuclei stained with Hoechst. Scale bars, 100 μm.

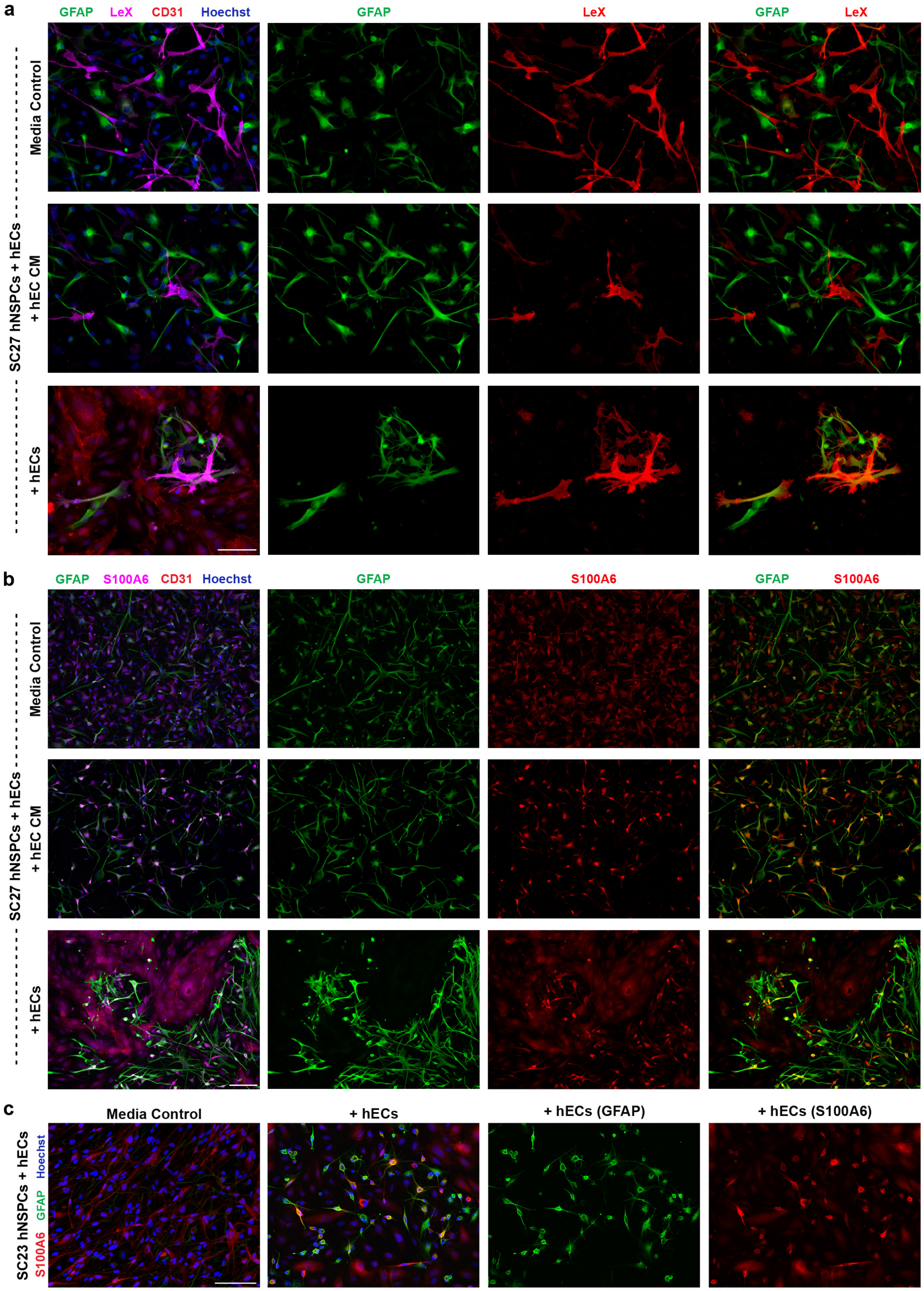

Supplementary Figure 3. hEC co-culture increases GFAP+LeX+ and GFAP+S100A6+

human type B cells (lower magnification and individual panel images related to Figure 2) **(a)** SC27 hNSPCs in media control, hEC CM, or with hECs stained for GFAP, LeX, and EC marker CD31. Panels with GFAP (green) and LeX (red) identify cells expressing both markers in hEC co-culture. **(b)** SC27 hNSPCs in media control, hEC CM, or + hECs immunostained for GFAP, S100A6, and EC marker CD31. Panels with GFAP (green) and S100A6 (red) identify cells expressing both markers in hEC co-culture. **(c)** SC23 hNSPCs in media control or + hECs immunostained for GFAP, S100A6, and EC marker CD31. Panels with GFAP (green) and S100A6 (red) identify cells expressing both markers in hEC co-culture. Co-cultures were grown for 5 days. Nuclei were stained with Hoechst. Scale bars, 100 μm.

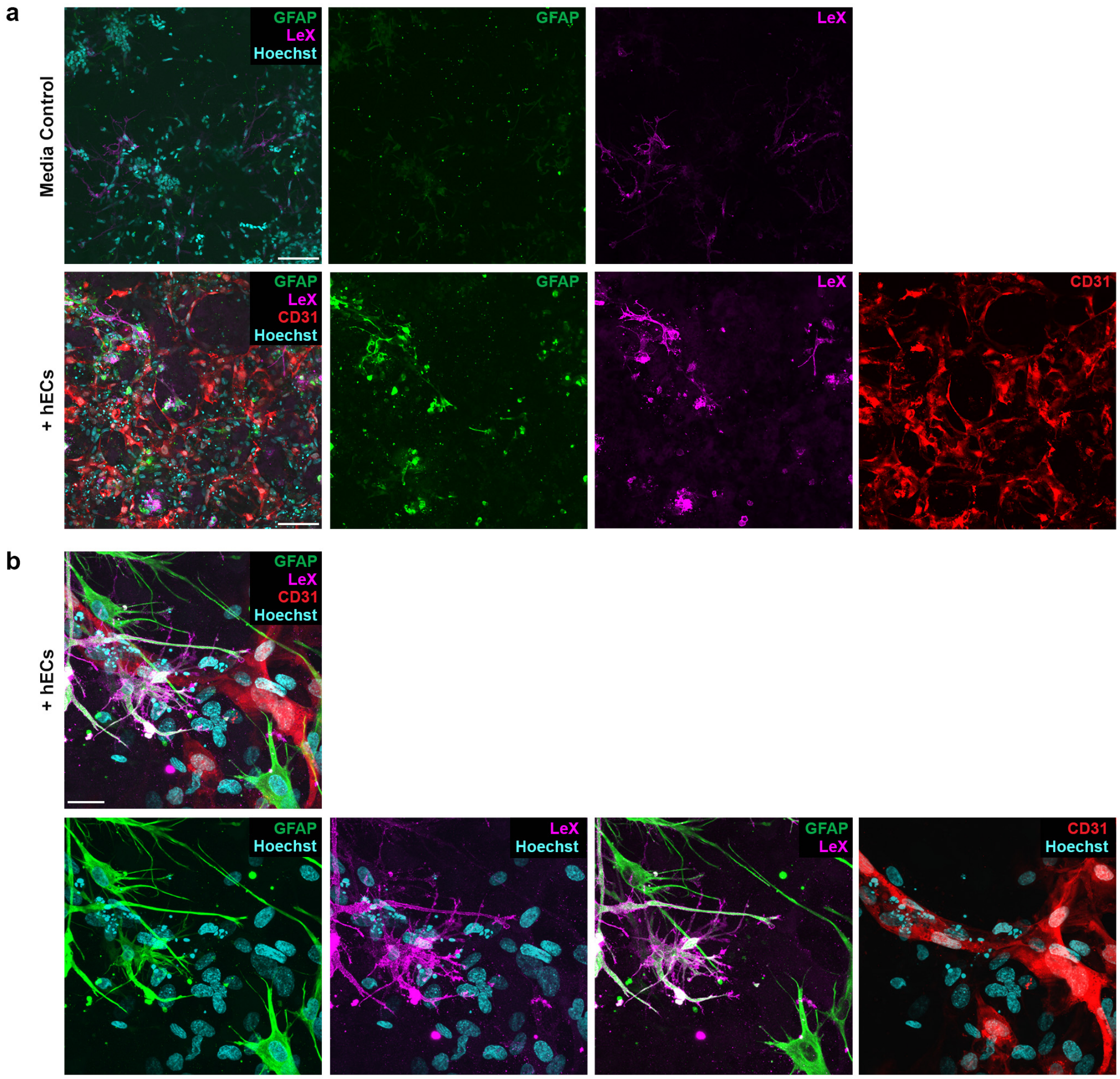

Supplementary Figure 4. Co-culture of hNSPCs and hECs in 3D scaffold increases GFAP+LeX+ human type B cells

(a) Cells were seeded in 3D scaffolds that mimic brain mechanical properties and ECM composition. SC27 hNSPCs were seeded alone (media control) or with hECs (+hECs), cultured for 5 days, and stained for GFAP, LeX, and EC marker CD31. Panels with GFAP and LeX identify hNSPCs expressing these markers in hEC co-culture by confocal microscopy. Scale bars, 100 µm. **(b)** High magnification images of SC27 hNSPCs seeded with hECs in 3D scaffolds show co-localization of GFAP and LeX and extension of hNSPC processes that contact vessels formed by hECs. Scale bar, 30 µm. All nuclei stained with Hoechst.

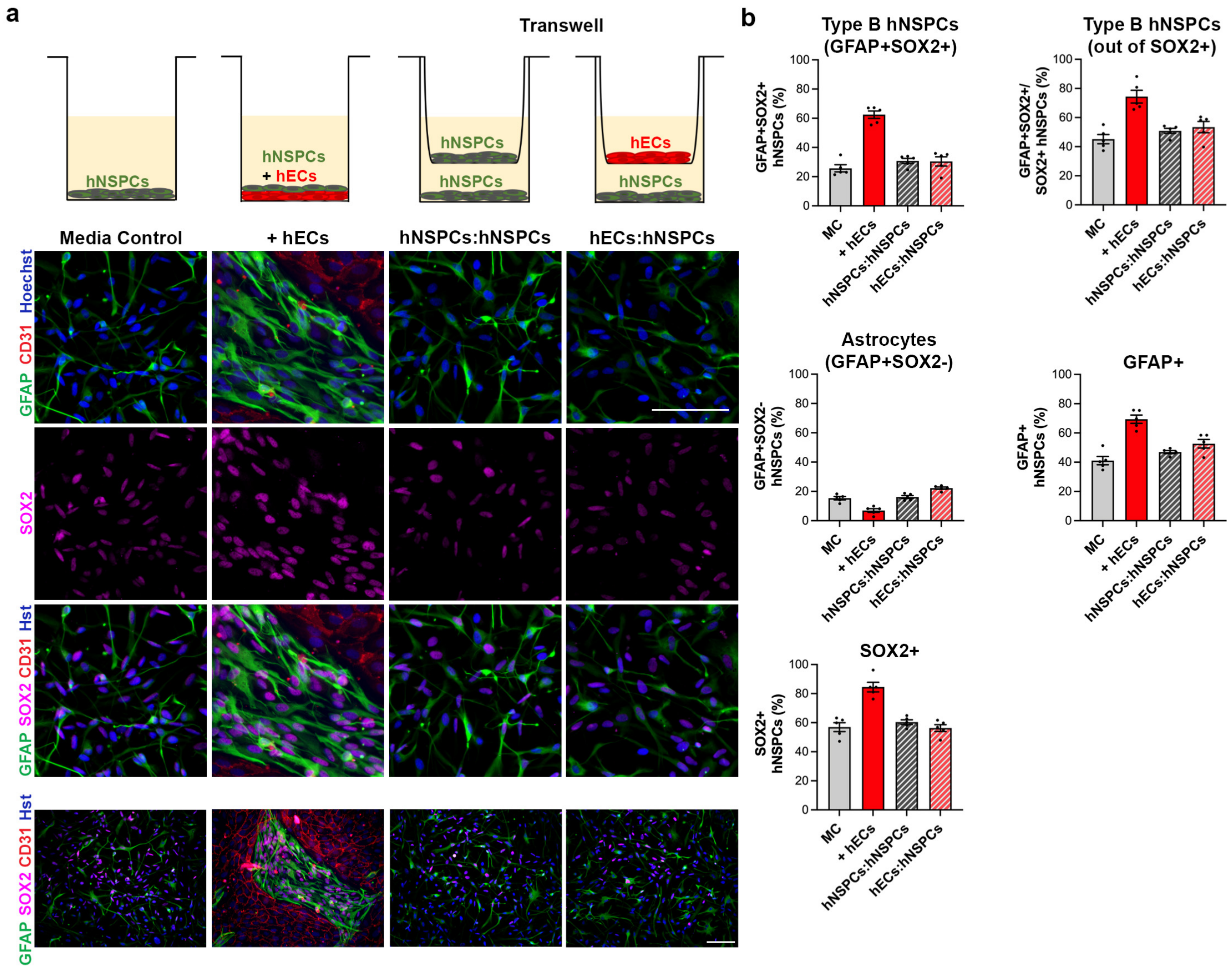

Supplementary Figure 5. Transwell study shows no effect of hEC secreted factors on hNSPC GFAP and SOX2 co-expression

(a) Schematic of the transwell experimental design showing mono-culture of hNSPCs, co-culture of hNSPCs and hECs, hNSPCs in the insert and the well (hNSPC:hNSPC), and hECs in the insert with hNSPCs in the well (hEC:hNSPC). Representative images below the schematic show cells plated in the wells in each of the conditions stained for GFAP, SOX2, and CD31. Images in bottom row are at lower magnification. **(b)** Quantification of the percentage of type B hNSPCs (GFAP+SOX2+), type B hNSPCs out of total number of SOX2+ hNSPCs, astrocytes (GFAP+SOX2-), GFAP+, and SOX2+ hNSPCs demonstrates an effect of hEC co-culture on all percentages while hEC transwell CM (hEC:hNSPC) had no effect compared to MC and hNSPC:hNSPC cultures. Nuclei stained with Hoechst. Scale bars, 100 µm, n=1 biological replicate, 5 areas quantified per sample, percentages are out of total hNSPCs, mean with SEM. Source data are provided as a Source Data file.

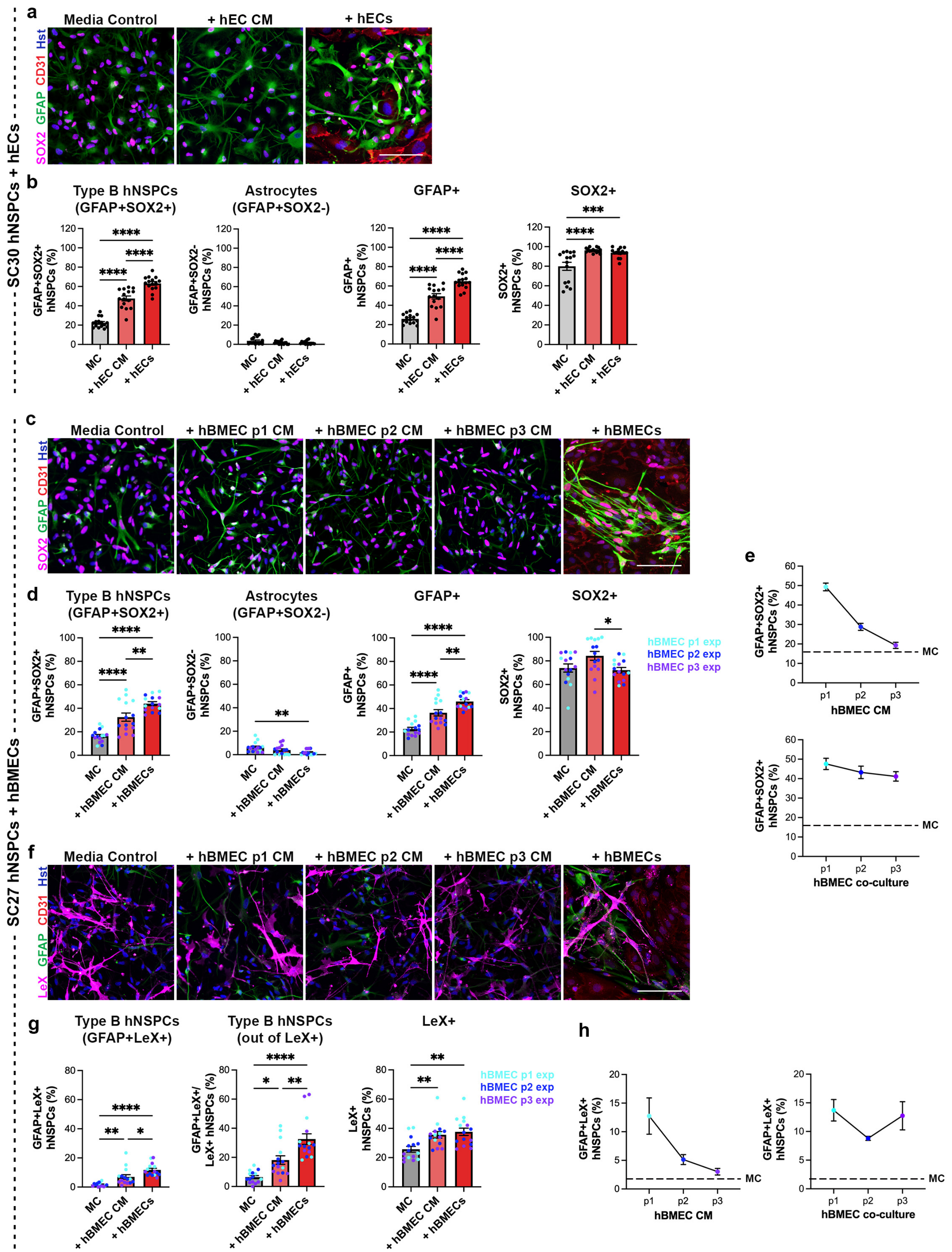

SC30 hNSPCs + hECs
a
Media Control
+ hEC CM
+ hECs
SOX2 GFAP CD31 Hst
b
Type B hNSPCs (GFAP+SOX2+)
Astrocytes (GFAP+SOX2-)
GFAP+
SOX2+
GFAP+SOX2+ hNSPCs (%)
GFAP+SOX2- hNSPCs (%)
GFAP+ hNSPCs (%)
SOX2+ hNSPCs (%)
MC
+ hEC CM
+ hECs
SC27 hNSPCs + hBMECs
c
Media Control
+ hBMEC p1 CM
+ hBMEC p2 CM
+ hBMEC p3 CM
+ hBMECs
SOX2 GFAP CD31 Hst
d
Type B hNSPCs (GFAP+SOX2+)
Astrocytes (GFAP+SOX2-)
GFAP+
SOX2+
hBMEC p1 exp
hBMEC p2 exp
hBMEC p3 exp
MC
+ hBMEC CM
+ hBMECs
e
GFAP+SOX2+ hNSPCs (%)
p1
p2
p3
hBMEC CM
hBMEC co-culture
MC
f
Media Control
+ hBMEC p1 CM
+ hBMEC p2 CM
+ hBMEC p3 CM
+ hBMECs
LeX GFAP CD31 Hst
g
Type B hNSPCs (GFAP+LeX+)
Type B hNSPCs (out of LeX+)
LeX+
GFAP+LeX+ hNSPCs (%)
GFAP+LeX+/ LeX+ hNSPCs (%)
LeX+ hNSPCs (%)
hBMEC p1 exp
hBMEC p2 exp
hBMEC p3 exp
h
GFAP+LeX+ hNSPCs (%)
hBMEC CM
hBMEC co-culture
MC

Supplementary Figure 6. Effect of hEC conditioned media varies with cell source
(a) SC30 hNSPCs in media control and hEC co-culture (+ hECs) as shown in Figure 1c, with the addition of hEC conditioned media (+ hEC CM) were immunostained for GFAP, SOX2, and CD31. **(b)** GFAP+SOX2+ type B cells are significantly greater in +hECs compared to MC (****p<0.0001) or hEC CM (****p<0.0001) and hEC CM is greater than MC (****p<0.0001). There was no significant formation of astrocytes by SC30 hNSPCs in any condition. GFAP+ cells are highest in +hEC compared to MC (****p<0.0001) or hEC CM (****p<0.0001) and hEC CM is greater than MC (****p<0.0001). SOX2+ cells are higher in +hEC compared to MC (***p=0.0009) and hEC CM compared to MC (****p<0.0001). **(c)** SC27 hNSPCs in MC and in co-culture with human brain microvascular ECs (+ hBMECs) as shown in Figure 1d, with the addition of hBMEC conditioned media collected from hBEMCs at passages 1-3 (+ hBMEC p1-3 CM), and immunostained for GFAP, SOX2, and CD31. **(d)** GFAP+SOX2+ hNSPCs significantly increased in hBMEC CM compared to MC (****p<0.0001) and in +hBMECs compared to MC (****p<0.0001). Co-culture with hBMECs significantly increased type B cells compared to hBMEC CM (**p=0.0038). GFAP+SOX2- astrocytes showed a significant decrease in hBMEC co-culture compared to MC (**p=0.0058). GFAP+ hNSPCs significantly increased in hBMEC CM (****p<0.0001) and in +hBMECs (****p<0.0001) compared to MC. Co-culture with hBMECs significantly increased GFAP+ cells compared to hBMEC CM (**p=0.0039). SOX2+ hNSPCs was higher in hBMEC CM compared to +hBMECs (*p=0.0287). There was no significant difference in SOX2+ hNSPCs in +hBMECs compared to MC (p=0.9169). MC and + hBMEC data are as shown in Figure 1d. **(e)** GFAP+SOX2+ type B cells decreased with increasing hBMEC passage number (p1-p3 as shown in d) for CM but remained relatively constant for hBMEC co-culture (n=1 at each passage). **(f)** SC27 hNSPCs in MC, +hBMEC p1-p3 CM, and + hBMEC co-culture were stained for GFAP, LeX, and CD31. **(g)** GFAP+LeX+ type B hNSPCs increased in +hBMECs (****p<0.0001) and hBMEC CM (**p=0.0052) compared to MC. Co-culture with hBMECs significantly increased type B cells compared to hBMEC CM (*p=0.0106). GFAP+LeX+ type B hNSPCs out of total number of LeX+ hNSPCs increased in +hBMECs (****p<0.0001) and hBMEC CM (*p=0.0130) compared to MC. Co-culture with hBMECs generated significantly more type B cells compared to hBMEC CM (**p=0.0019). LeX+ hNSPCs increased in hBMEC CM (**p=0.0098) and +hBMECs (**p=0.0016) compared to MC. **(h)** GFAP+LeX+ cells decreased with increasing hBMEC passage number (p1-p3 as shown in g) for CM but did not show a consistent decrease for hBMEC co-culture (n=1 at each passage). Dot color represents data from hBMEC experiments at different passages: p1, p2, and p3. Dashed line represents MC mean. Nuclei stained with Hoechst. Scale bars, 100 µm, n=3 independent biological replicates unless otherwise specified, 5 areas quantified per sample, percentages are out of total hNSPCs unless otherwise specified, mean with SEM. Analysis used one-way ANOVA with Tukey post-hoc test for multiple comparisons. Source data are provided as a Source Data file.

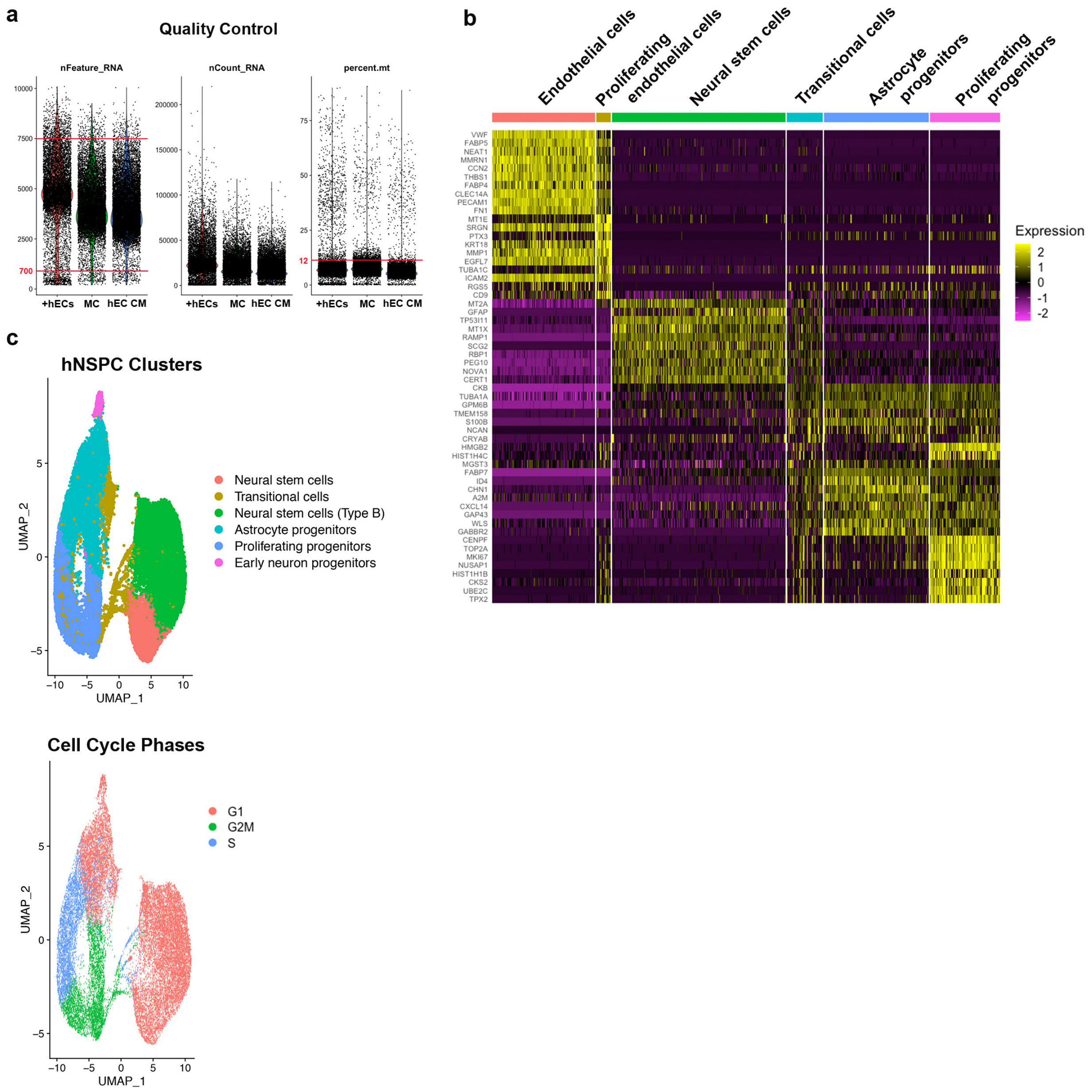

Supplementary Figure 7. Single-cell RNA sequencing quality control and cluster analysis. **(a)** Violin plots show the distribution of scRNAseq datasets in terms of the number of genes (nFeature_RNA), number of RNA molecules (nCount_RNA), and percent mitochondrial genes (percent_mt) of cells in each scRNAseq dataset: co-cultures (+hECs), hNSPCs in media control (MC), and in hEC conditioned media (hEC CM). Red lines represent cut-offs used for quality control. Cells with nFeatures of more than 700 and less than 7500, and with less than 12% mitochondrial genes were used for analysis. **(b)** Heatmap of the top 10 differentially expressed genes in each hEC or hNSPC cluster depicted in Figure 3a based on average log2 fold change. Yellow represents high expression and purple represents low expression. **(c)** UMAP plot of merged datasets of hNSPC clusters from media control, hEC conditioned media, and hEC co-cultures. A corresponding UMAP shows cell cycle analysis with colors representing the G1, G2/M, and S phases of the cell cycle. Neural stem cells (Type B), neural stem cells, astrocyte

progenitors, and early neuron progenitors are primarily in G1 while proliferating progenitors and some transitional cells are in G2/M and S phases of the cell cycle.

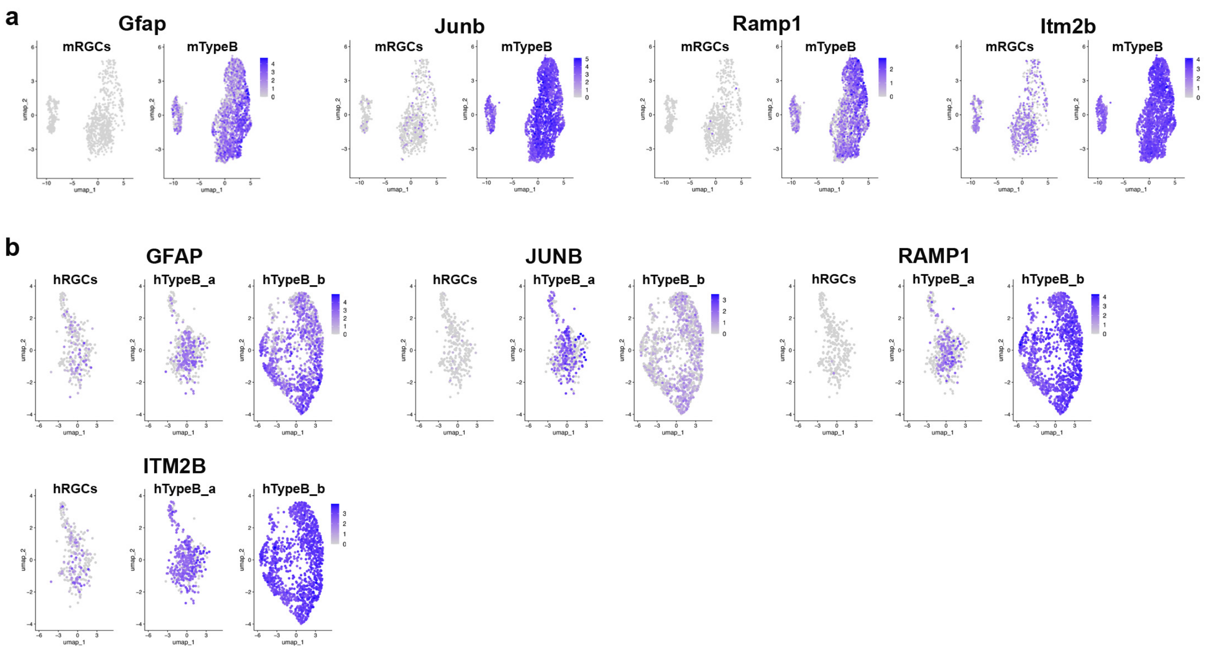

Supplementary Figure 8. Genes expressed by mouse and human type B cells. UMAP plots of integrated datasets[1-4] from Figure 4 demonstrate genes expressed by **(a)** mouse type B cells (mTypeB) and **(b)** human type B cells (hTypeB) including our type B hNSPCs (hTypeB_b). These genes are not expressed or expressed at lower levels by mouse RGCs (mRGCs) or human RGCs (hRGCs).

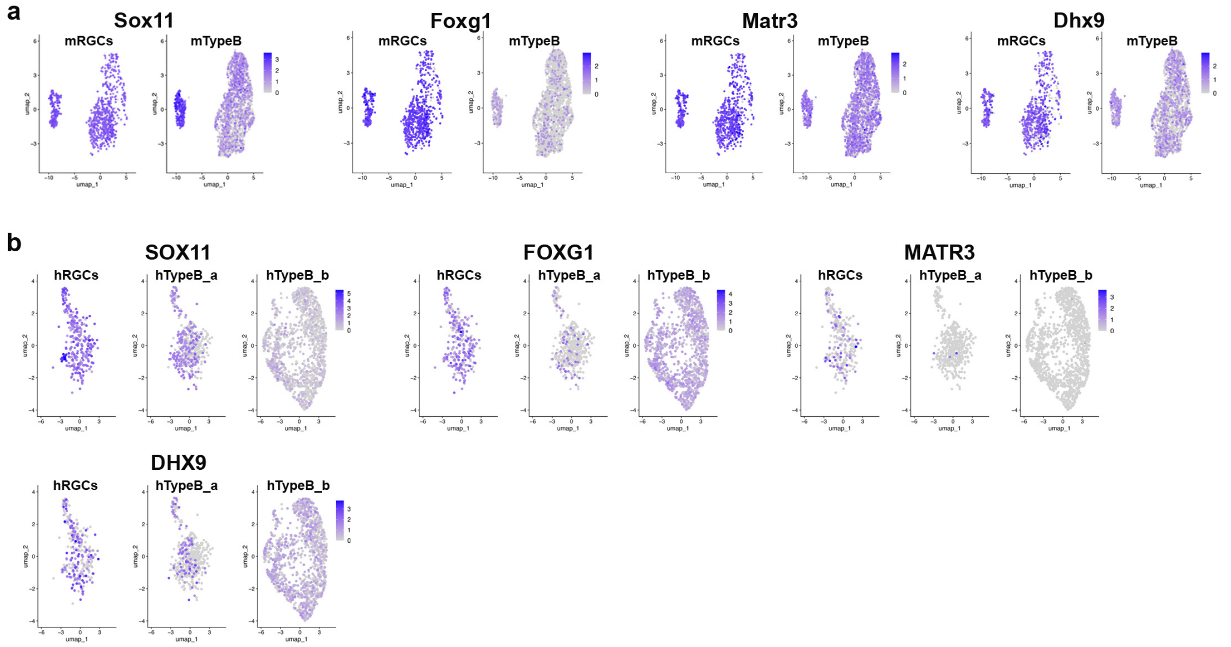

Supplementary Figure 9. Genes expressed by mouse and human radial glial cells. UMAP plots of integrated datasets[1-4] from Figure 4 demonstrate genes expressed by **(a)** mouse radial glial cells (mRGCs) and **(b)** human radial glial cells (hRGCs). These genes are expressed at lower levels by mouse type B cells (mTypeB) or human type B cells (hTypeB).

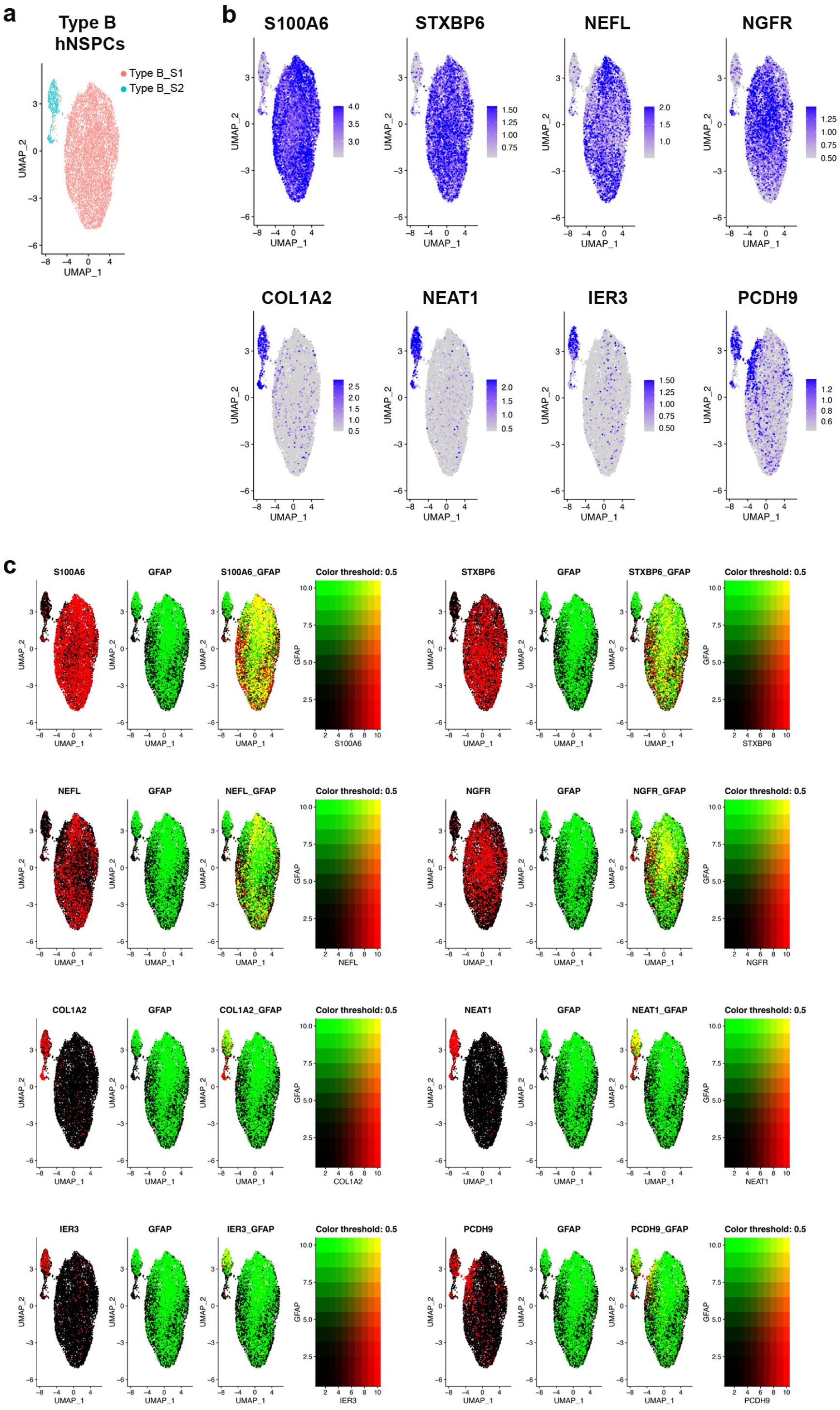

d

Gene	% Type B hNSPCs expressing gene	% TypeB hNSPCs co-expressing GFAP and gene
GFAP	87.77	
S100A6	97.91	86.08
STXBP6	77.58	68.28
NEFL	57.43	50.68
NGFR	68.37	60.92
COL1A2	11.02	8.88
NEAT1	15.36	13.71
IER3	9.00	8.24
PCDH9	27.90	24.81

Supplementary Figure 10. Type B hNSPC subclusters.
(a) UMAP of type B hNSPC subset analysis revealing two type B subclusters, type B subcluster 1 (Type B_S1) and the smaller type B subcluster 2 (Type B_S2). **(b)** Plots reveal higher expression of S100A6, STXBP6, NEFL, and NGFR in Type B_S1 and higher expression of COL1A2, NEAT1, IER3, and PCDH9 in Type B_S2. **(c)** Plot showing expression of subcluster

genes from b (red), GFAP (green), and co-expression (yellow), color threshold set to 0.5. Heatmaps in arbitrary units. For plots in (b) and (c), a minimum and maximum cutoff value for each feature was set at quantiles 10 and 90, respectively. (d) Table demonstrates the percentage of type B hNSPCs expressing GFAP and subcluster genes and the percentage co-expressing both GFAP and subcluster genes. Cells with gene expression > 0 were counted as positive.

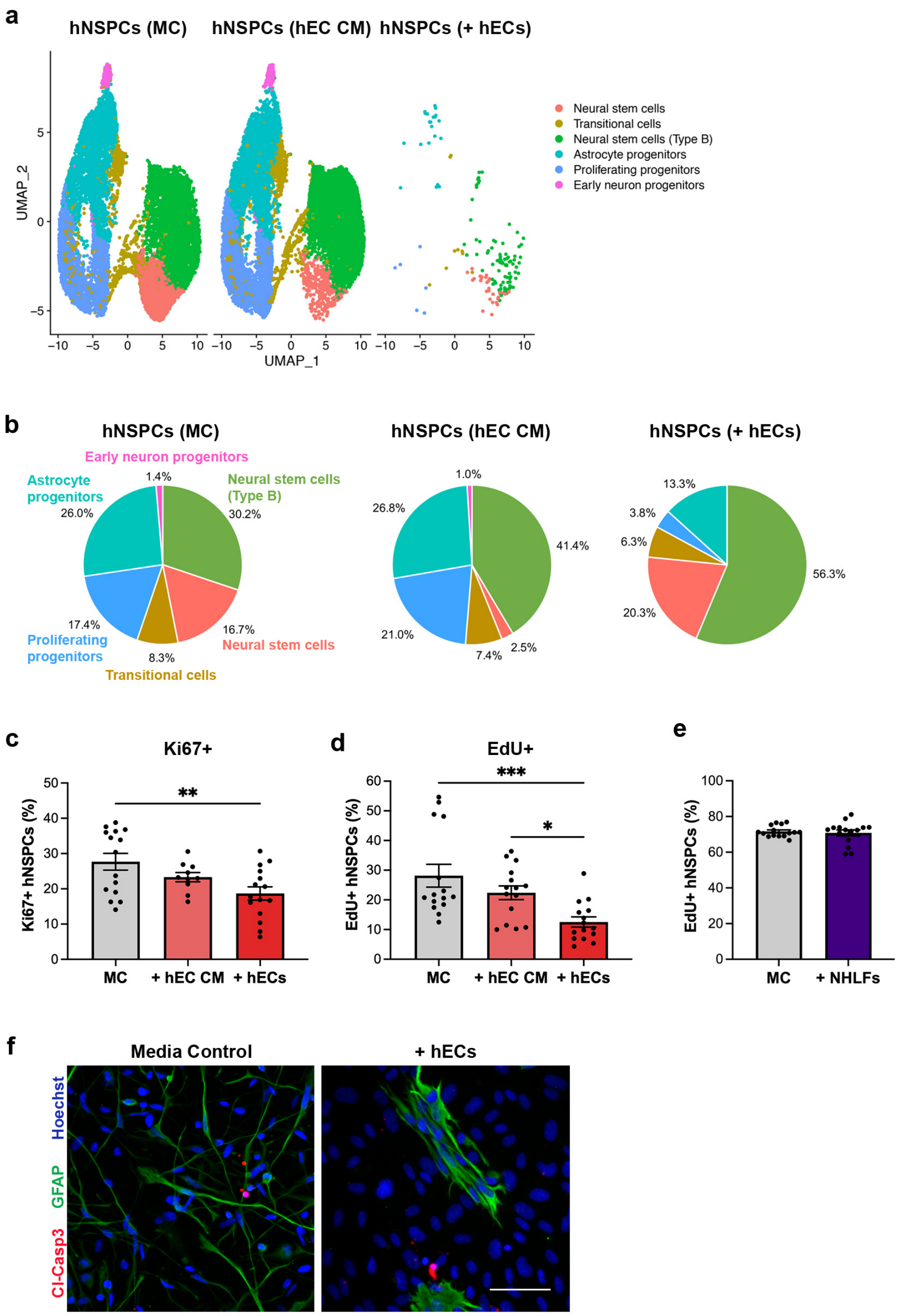

Supplementary Figure 11. Effect of hEC conditioned media on hNSPC population composition.

(a) UMAP from scRNAseq analysis shown in Figure 3c split by conditions; hNSPCs in media control (MC), hNSPCs in conditioned media (hEC CM), and hNSPCs in co-culture (+hECs). There are fewer hNSPCs in the co-culture condition compared to controls due to the co-culture ratio of 1 hNSPC to 5 hECs and the higher hEC proliferation rate compared to hNSPCs. The lower number of hNSPCs does not significantly affect cluster identification as merged datasets were used to identify clusters and the number of cells (158 hNSPCs) is sufficient to determine the proportion in different clusters. **(b)** Pie chart demonstrating the percentage of cells in each

cluster out of the total number of hNSPCs in each condition. Percentages calculated from scRNAseq data. **(c)** Percentage of Ki67+ hNSPCs was significantly lower in hEC co-culture (+hECs) compared to media control (MC) (**p=0.0059). HEC CM (n=2, excluded from statistical analysis) did not have the same effect as + hECs. Data for the MC and +hEC conditions is from Figure 5d and included for comparison. (**d)** Percentage of EdU+ hNSPCs was significantly lower in hEC co-culture compared to MC (***p=0.0008) and hEC CM (*p=0.0408). There was no significant difference in EdU+ hNSPCs in CM compared to MC (p=0.3224). Data for the MC and +hEC conditions is from Figure 5e. **(e)** Percentage of EdU+ hNSPCs did not differ in NHLF co-culture compared to MC (n=1, 15 areas quantified per sample). **(f)** SC27 hNSPCs were grown in MC or hEC co-culture and stained for GFAP and cleaved-caspase 3 (Cl-Casp3) apoptosis marker. Very few Cl-Casp3 positive cells were detected in either condition. Nuclei stained with Hoechst. Scale bars, 100 μm, n=3 independent biological replicates unless otherwise noted, 5 areas quantified per sample, percentages are out of total hNSPCs, mean with SEM. Analysis used (c) unpaired two-tailed Student's t-test and (d) one-way ANOVA with Tukey post-hoc test for multiple comparisons**.** Source data are provided as a Source Data file.

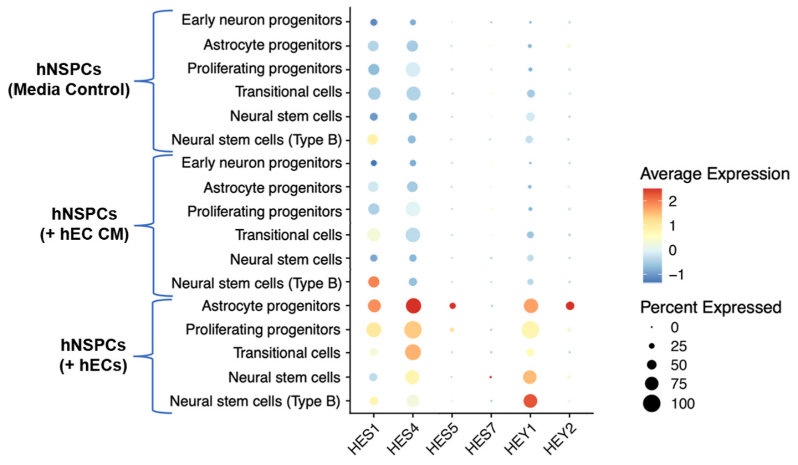

Supplementary Figure 12. hEC co-culture upregulates hNSPC expression of Notch signaling genes while hEC secreted factors have no effect.
Dot plot of the expression of Notch downstream mediators by cells in each hNSPC cluster in MC, +hEC CM, and +hECs conditions. The average gene expression of cells in the cluster compared to the average expression across all clusters is noted by the color. The size of the dot represents the percent of cells in the clusters expressing the marker of interest.

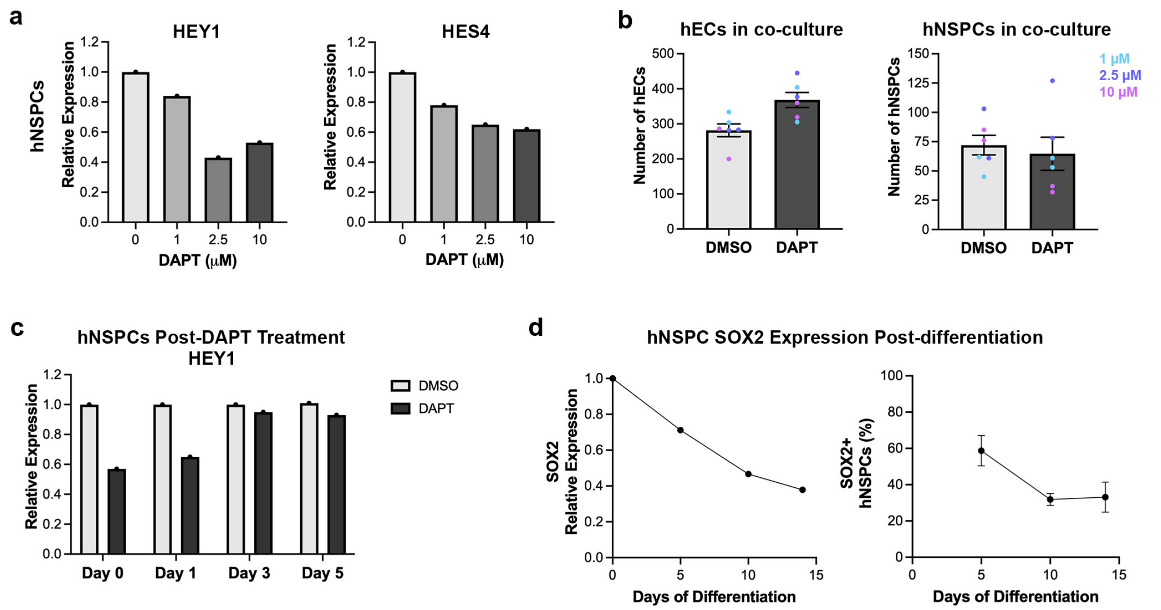

Supplementary Figure 13. DAPT reduces the expression of Notch downstream mediators, and this effect lasts for one day after treatment.

(a) SC27 hNSPCs treated with varying concentrations of the Notch inhibitor, DAPT, exhibited lower HEY1 and HES4 Notch downstream mediator compared to untreated hNSPCs (0 μM), n=1 biological replicate. **(b)** Treatment of co-cultures with 1 μM, 2.5 μM, and 10 μM DAPT caused an increase in the number of CD31+ hECs at all concentrations. The number of hNSPCs (non-CD31+) did not change at 1 μM and 2.5 μM while 10 μM DAPT seemed to decrease hNSPC number in co-culture, n=1 biological replicate. **(c)** HNSPCs treated with 10 μM DAPT demonstrated a decrease in HEY1 expression for one day after treatment, n=1 biological replicate. **(d)** SOX2 expression slowly decreases during hNSPC differentiation, as shown by qRT-PCR and relative to undifferentiated hNSPCs (t=0), n=1 biological repeat, and immunocytochemistry for SOX2, n=3 biological repeats. Relative expression determined via qRT-PCR normalized to (a, c) GAPDH or (d) 18S. Graphs show mean with SEM. Source data are provided as a Source Data file.

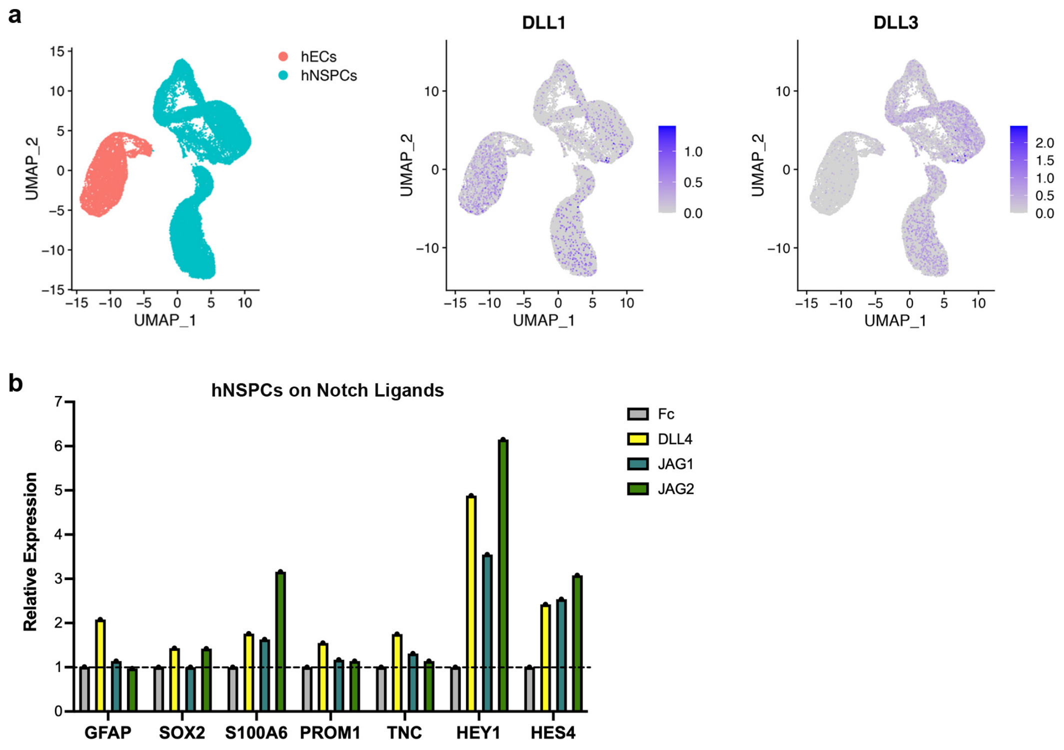

Supplementary Figure 14. Notch ligand expression by hECs and their effect on hNPSC type B cell marker expression.
(a) UMAP demonstrating hEC and hNSPC cell types. Featureplots reveal low expression of DLL1 and DLL3 Notch ligands by hECs. **(b)** HNSPCs (SC27) plated on coverslips coated with DLL4, JAG1, or JAG2 for 5 days demonstrate an increase in HEY1 and HES4 expression compared to Fc control by qRT-PCR normalized to GAPDH. GFAP, SOX2, S100A6, PROM1, and TNC expression increased with DLL4. Graphs show mean, n=1 biological replicate. Source data are provided as a Source Data file.

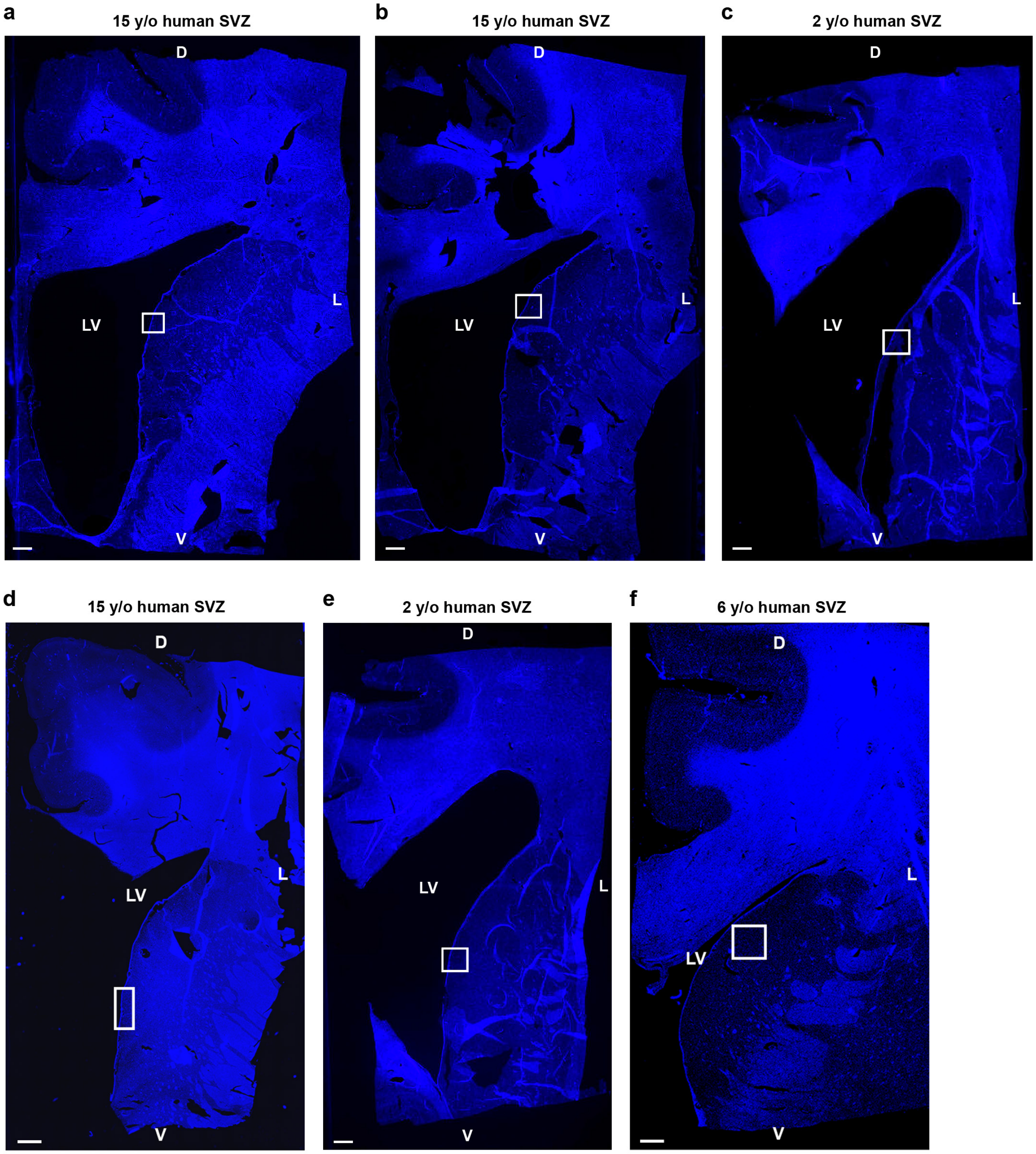

Supplementary Figure 15. 4x images of the human SVZ show anatomical location for images in Figures 8, 9, and 10.
(a, b) Images of 15-year-old human brain tissue (4x) were tiled in order to show anatomical structures. White boxes denote region for images shown in Figure 8a and 8b, respectively. **(c)** Image of 2-year-old human brain tissue with white box denoting location of images in Figure 9b. **(d)** Image of 15-year-old human brain tissue shows anatomical structures. White box denotes region for images in Figure 10a. **(e)** Image of 2-year-old human brain tissue shows anatomical structures and white box denotes region for images in Figure 10b. **(f)** Image of 6-year-old human brain tissue with white box denoting location of images in Figure 10c. Dorsal (D), ventral

(V) and lateral (L) markers give orientation and LV designates the lateral ventricle; nuclei were stained with DAPI. Scale bars 2 mm.

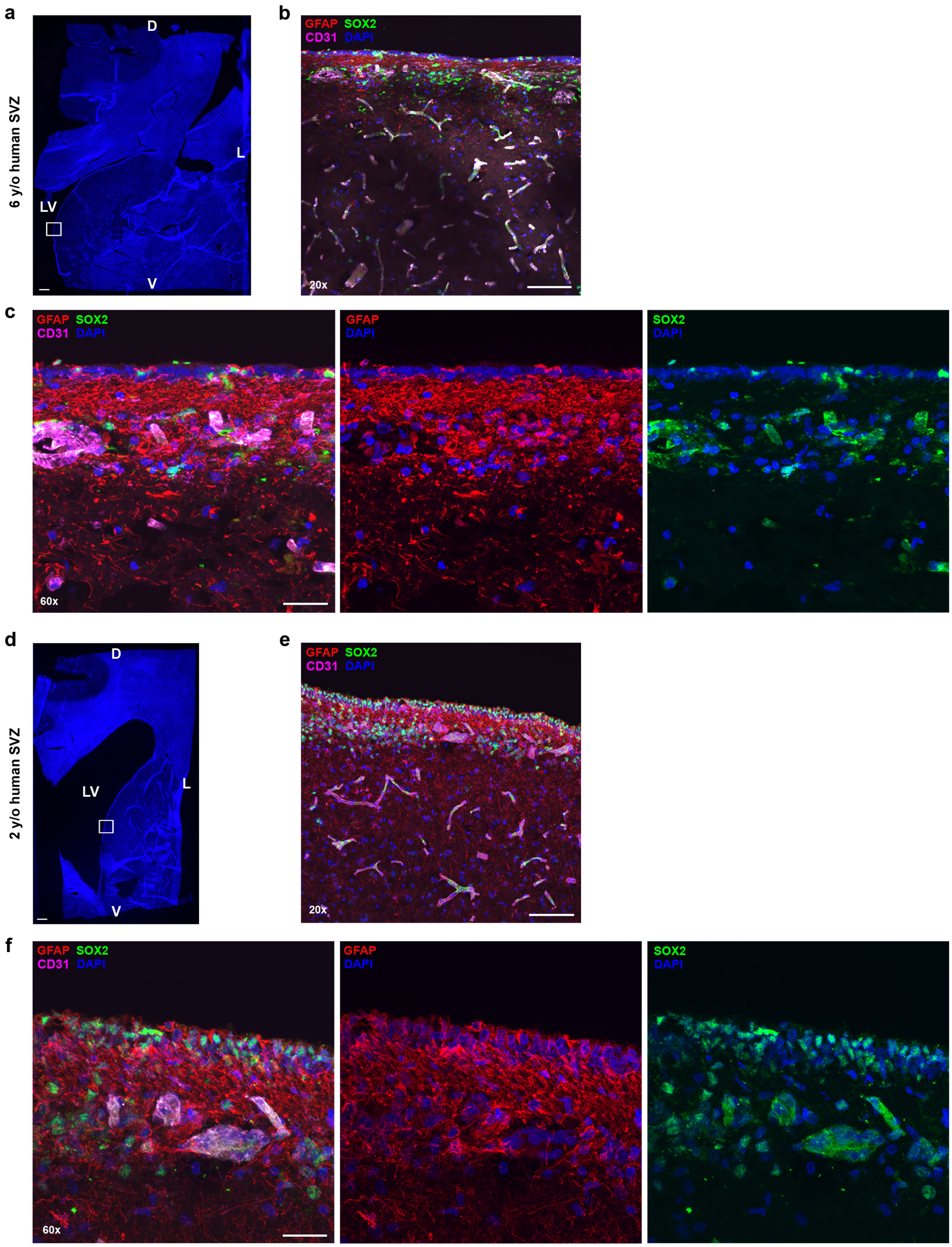

Supplementary Figure 16. Co-expression of type B hNSPC markers GFAP and SOX2 in the human SVZ at multiple stages.

(a) Images of 6-year-old human SVZ at 4x stained with DAPI were tiled to show anatomical structures and white box denotes region for images shown in b and c. Dorsal (D), ventral (V) and lateral (L) markers give orientation and LV designates the lateral ventricle. Scale bar 2 mm. **(b)** Lower magnification image (20x, scale bar 100 μm) of 6-year-old human SVZ stained for GFAP, SOX2, CD31 and DAPI and imaged by confocal microscopy shows enrichment of signal in the SVZ. **(c)** Higher magnification images (60x, scale bar 30 μm) of 6-year-old human SVZ show positively stained cells in the SVZ. **(d)** Images of 2-year-old human SVZ at 4x stained with DAPI were tiled to show anatomical structures and white box denotes region for images shown in e and f. Scale bar 2 mm. **(e)** Lower magnification image (20x, scale bar 100 μm) of 2-year-old human SVZ stained for GFAP, SOX2, CD31 and DAPI and imaged by confocal microscopy show enrichment of signal in the SVZ. **(f)** Higher magnification images (60x, scale bar 30 μm) of 2-year-old human SVZ show positively stained cells in the SVZ.

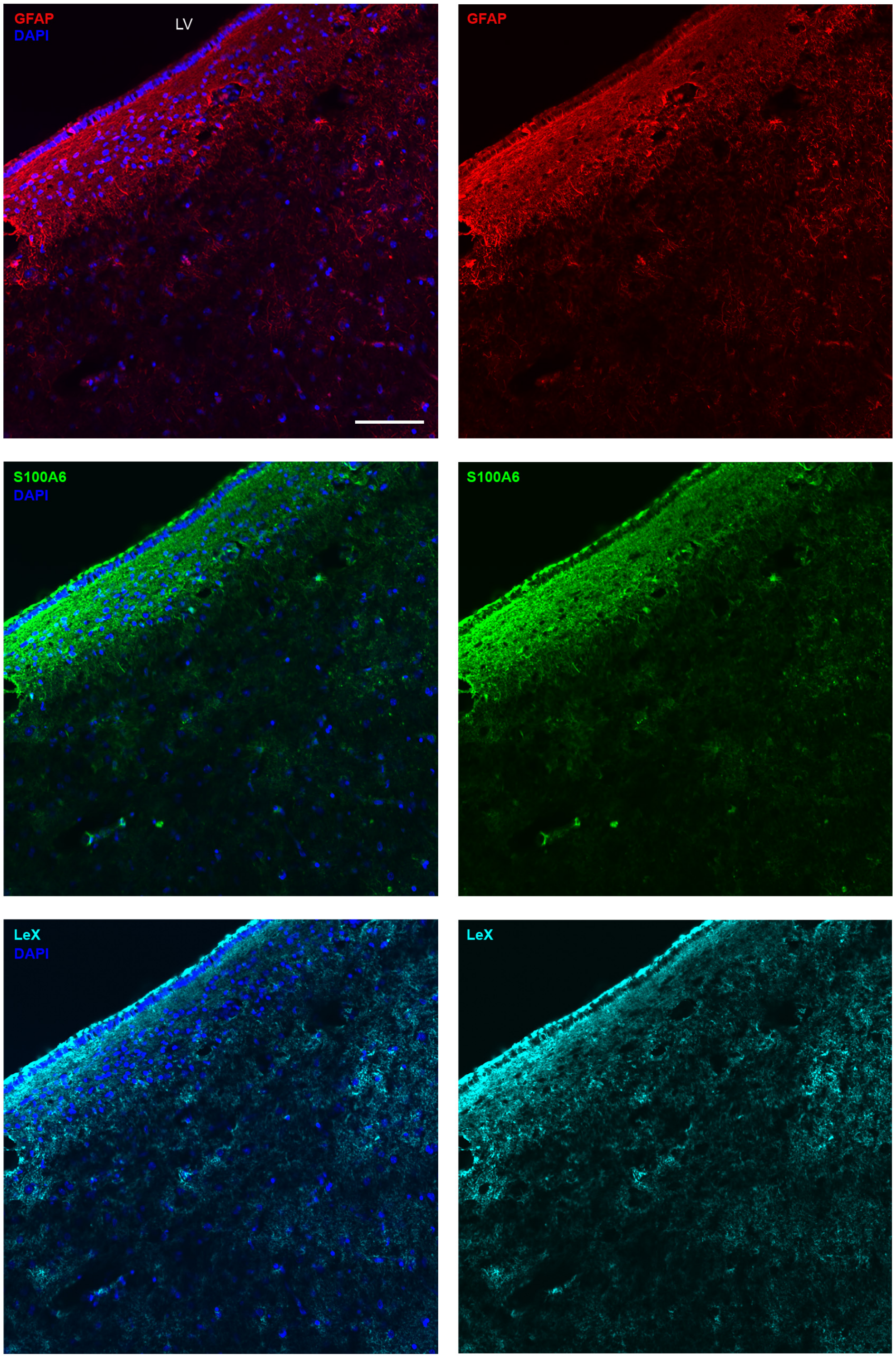

Supplementary Figure 17 (related to 8b). Co-expression of type B hNSPC markers GFAP, S100A6, and LeX in the human SVZ.
Low magnification (20x) confocal microscopy individual images of 15-year-old human SVZ stained for GFAP, S100A6, LeX and DAPI show co-localization in the SVZ. Scale bar 100 μm.

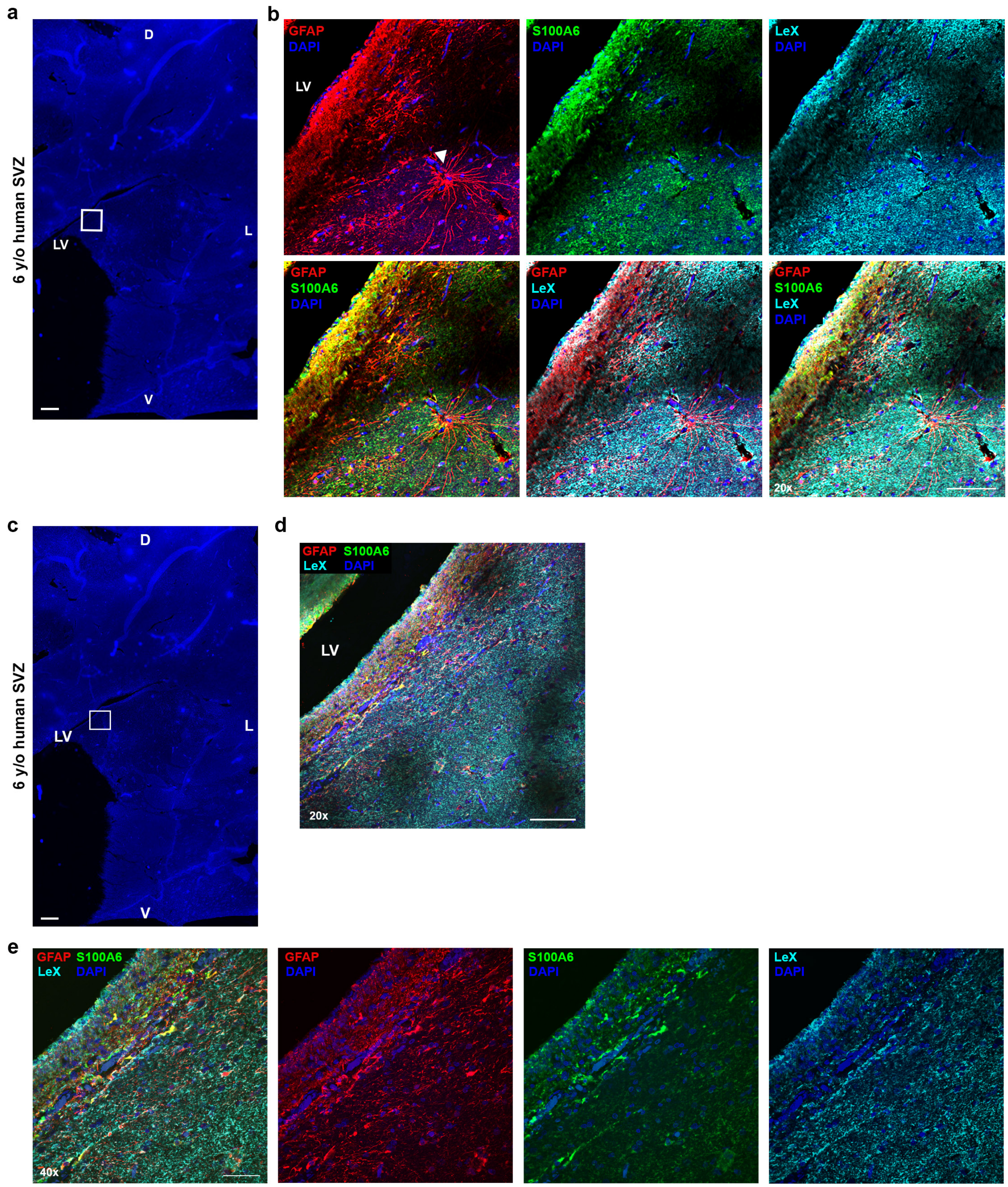

Supplementary Figure 18. Co-expression of type B hNSPC markers GFAP, S100A6, and LeX in the human SVZ at multiple stages (6 year old).
(a) Images of 6-year-old human SVZ at 4x stained with DAPI were tiled to show anatomical structures and white box denotes region for images shown in b. Dorsal (D), ventral (V) and lateral (L) markers give orientation and LV designates the lateral ventricle. Scale bar 2 mm. **(b)** Lower magnification confocal microscopy images (20x, scale bar 100 μm) of 6-year-old human

SVZ stained for GFAP, S100A6, LeX and DAPI show enrichment of signal in the SVZ. A prominent GFAP+ astrocyte with multiple processes away from the ventricle (designated by arrowhead) is not stained by S100A6 or LeX. **(c)** Images of 6-year-old human SVZ at 4x stained with DAPI with white box to denote region for images in d and e. Scale bar 2 mm. **(d)** Lower magnification confocal microscopy image (20x, scale bar 100 μm) of 6-year-old human SVZ stained for GFAP, S100A6, LeX and DAPI show enrichment of signal in the SVZ. **(e)** Higher magnification images (40x, scale bar 50 μm) of 6-year-old human SVZ show positively stained cells in the SVZ.

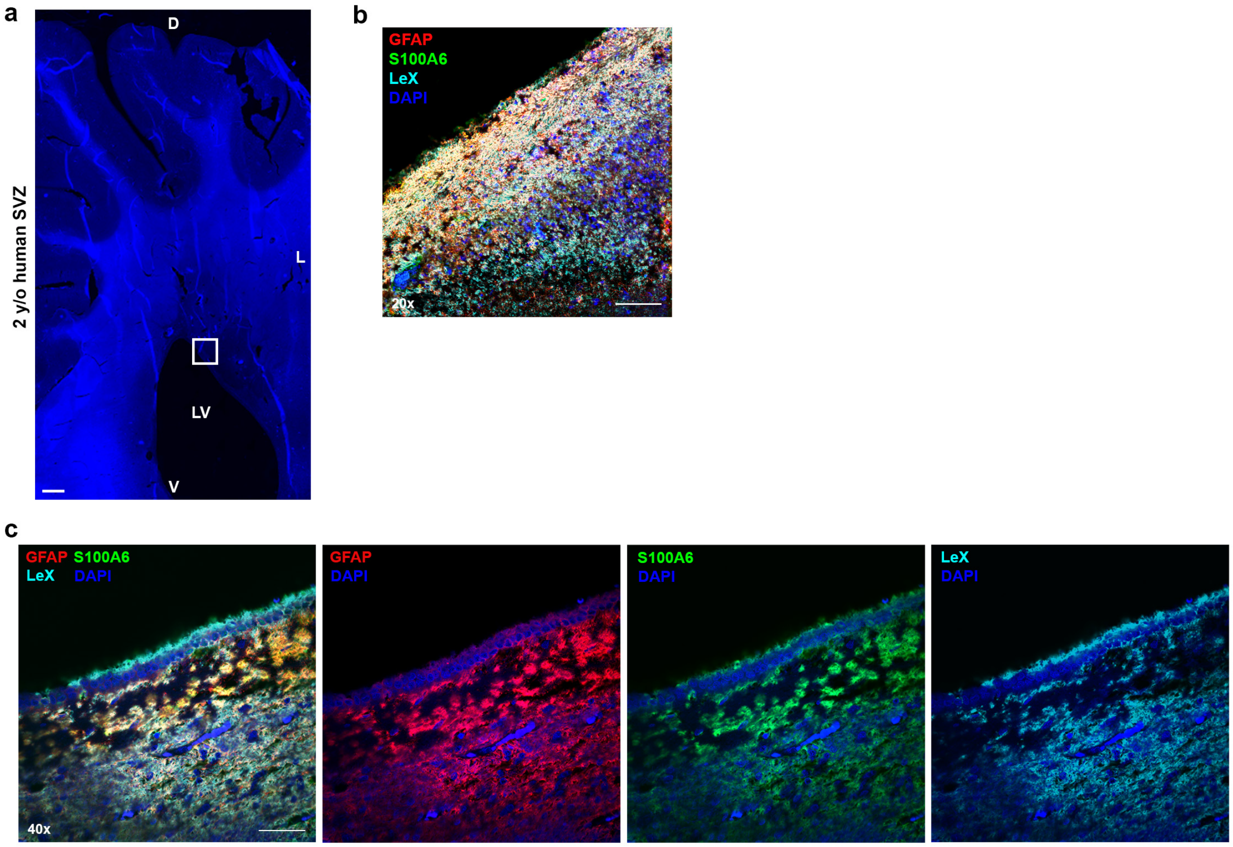

Supplementary Figure 19. Co-expression of type B hNSPC markers GFAP, S100A6, and LeX in the human SVZ at multiple stages (2 year old).
(a) Images of 2-year-old human SVZ at 4x stained with DAPI were tiled to show anatomical structures and white box denotes region for images shown in b and c. Dorsal (D), ventral (V) and lateral (L) markers give orientation and LV designates the lateral ventricle. Scale bar 2 mm.
(b) Lower magnification confocal microscopy images (20x, scale bar 100 μm) of 2-year-old human SVZ stained for GFAP, S100A6, LeX and DAPI show enrichment of signal in the SVZ.
(c) Higher magnification images (40x, scale bar 50 μm) of 2-year-old human SVZ show positively stained cells in the SVZ. The tissue is somewhat damaged immediately under the ependymal cell layer, but cells co-expressing markers are visible in SVZ tissue beneath the damaged region.

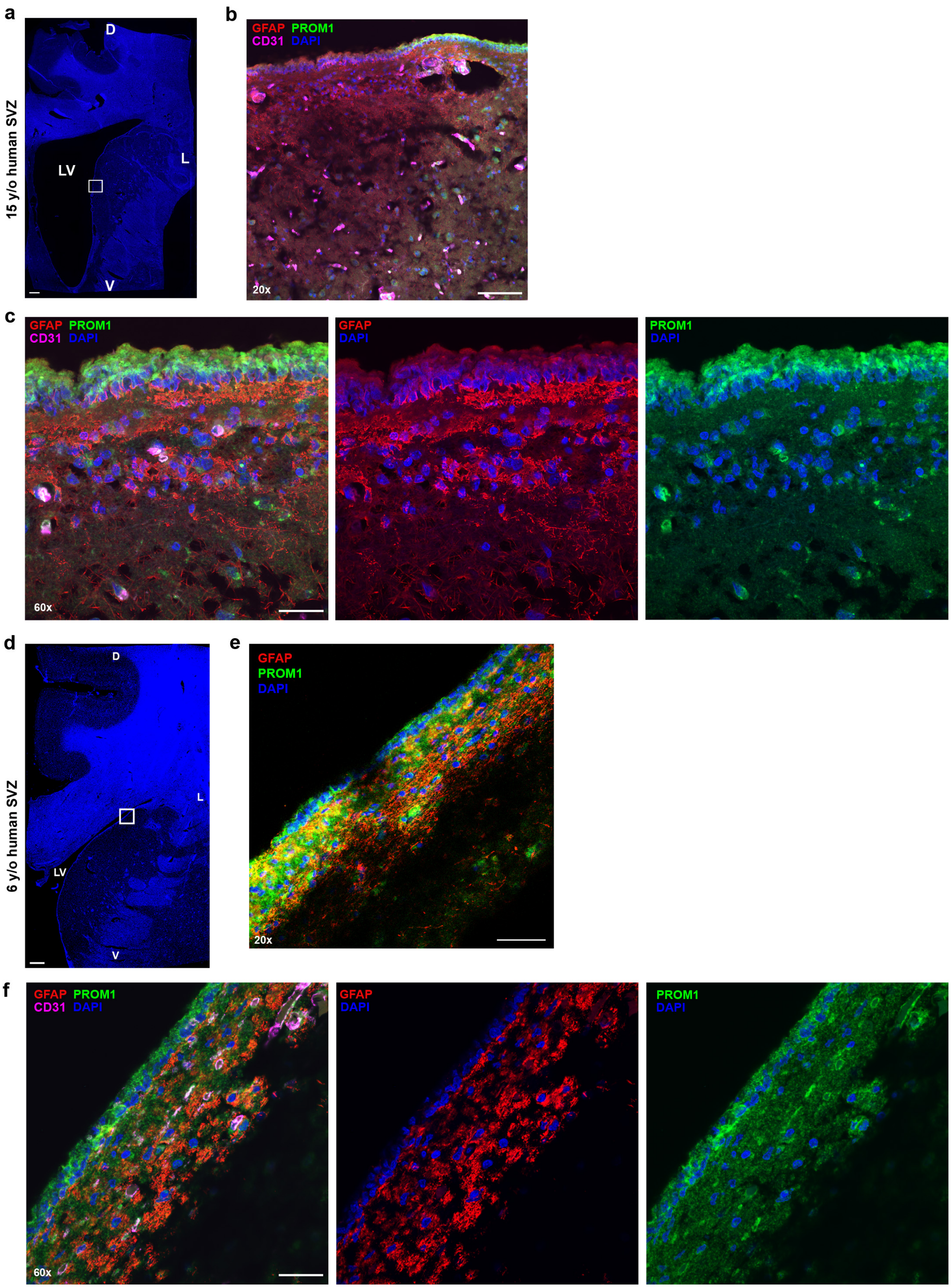

Supplementary Figure 20. Co-expression of type B hNSPC markers GFAP and PROM1 in

the human SVZ at multiple stages (15 year old, 6 year old).
(a) Images of 15-year-old human SVZ at 4x stained with DAPI were tiled to show anatomical structures and white box denotes region for images shown in b and c. Dorsal (D), ventral (V) and lateral (L) markers give orientation and LV designates the lateral ventricle. Scale bar 2 mm. **(b)** Lower magnification confocal microscopy image (20x, scale bar 100 μm) of 15-year-old human SVZ stained for GFAP, PROM1, CD31 and DAPI shows enrichment of signal in the SVZ. **(c)** Higher magnification images (60x, scale bar 30 μm) of 15-year-old human SVZ show positively stained cells in the SVZ. **(d)** Images of 6-year-old human SVZ at 4x stained with DAPI were tiled to show anatomical structures and white box denotes region for images shown in e and f. Scale bar 2 mm. **(e)** Lower magnification confocal microscopy image (20x, scale bar 100 μm) of 6-year-old human SVZ stained for GFAP, PROM1, and DAPI show enrichment of signal in the SVZ. **(f)** Higher magnification images (60x, scale bar 30 μm) of 6-year-old human SVZ show positively stained cells in the SVZ.

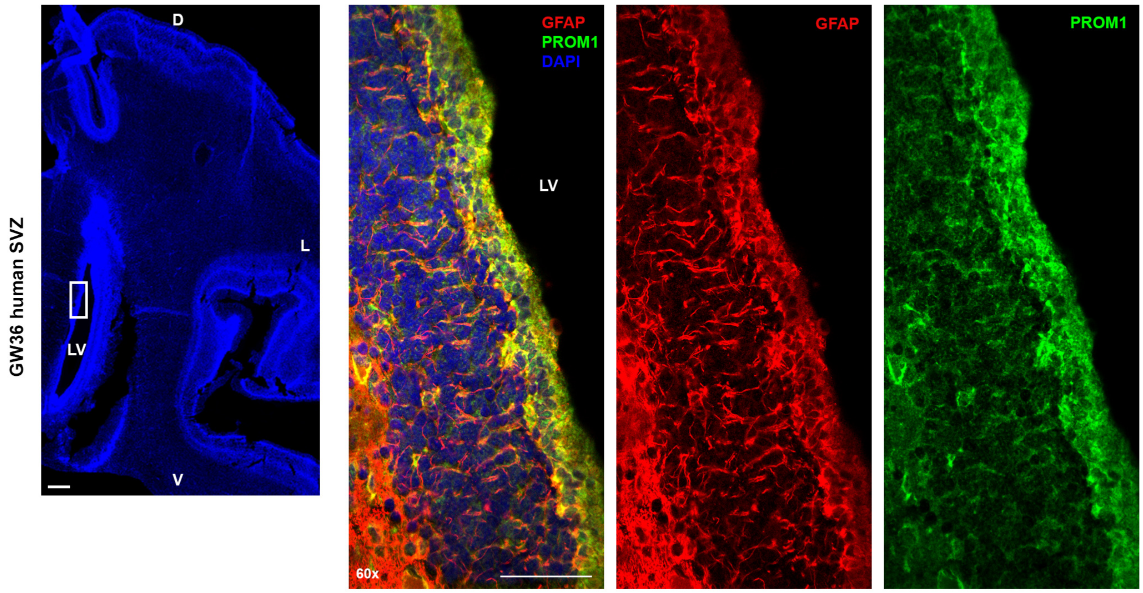

Supplementary Figure 21. Co-expression of type B hNSPC markers GFAP and PROM1 in the human SVZ at multiple stages (GW36).
Gestational week 36 (GW36) human SVZ imaged at 4x with DAPI to show location (scale bar 2 mm). White boxes in the 4x image denotes region for zoomed images. Dorsal (D), ventral (V) and lateral (L) markers give orientation and LV designates the lateral ventricle. GW36 human SVZ was stained for GFAP, PROM1 and DAPI and imaged by confocal microscopy (scale bar 100 μm).

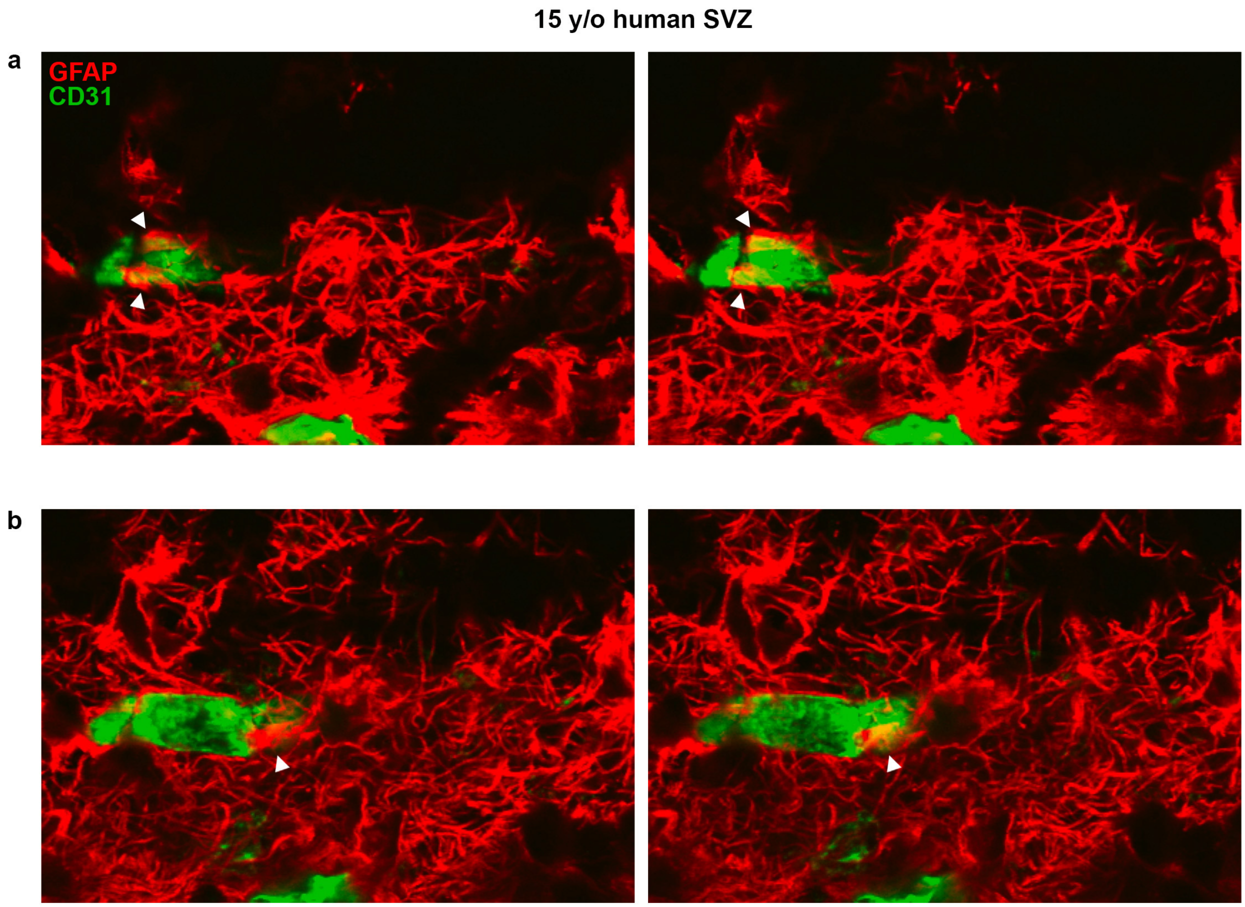

Supplementary Figure 22. GFAP+ processes contact vessels in the human SVZ; related to Figure 10a and Supplementary Movie 1.
Still frames from Supplementary Movie 1 of 15-year-old human SVZ stained for GFAP and CD31 show a vessel coursing through the SVZ in sequential frames at two locations (a and b) and arrowheads highlight points of contact with GFAP+ processes.

6 y/o human SVZ

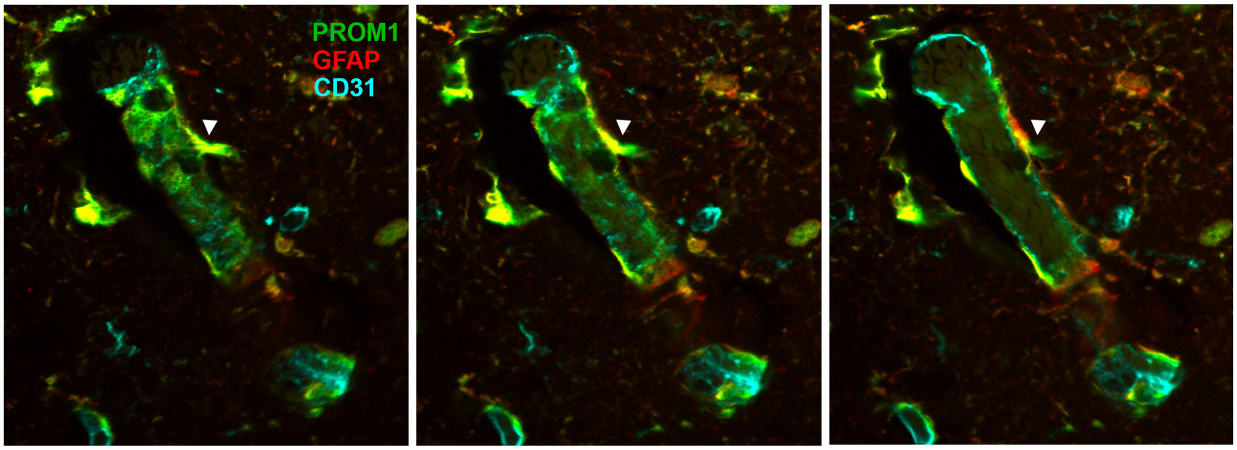

Supplementary Figure 23. GFAP+ and PROM1+ processes wrap around vasculature in the 6-year-old human brain; related to Figure 10c and Supplementary Movie 2.
Still frames from Supplementary Movie 2 of 6-year-old human SVZ stained for GFAP, PROM1 and CD31 show a CD31+ vessel contacted by a GFAP+PROM1+ process highlighted by arrowheads.

6 y/o human SVZ

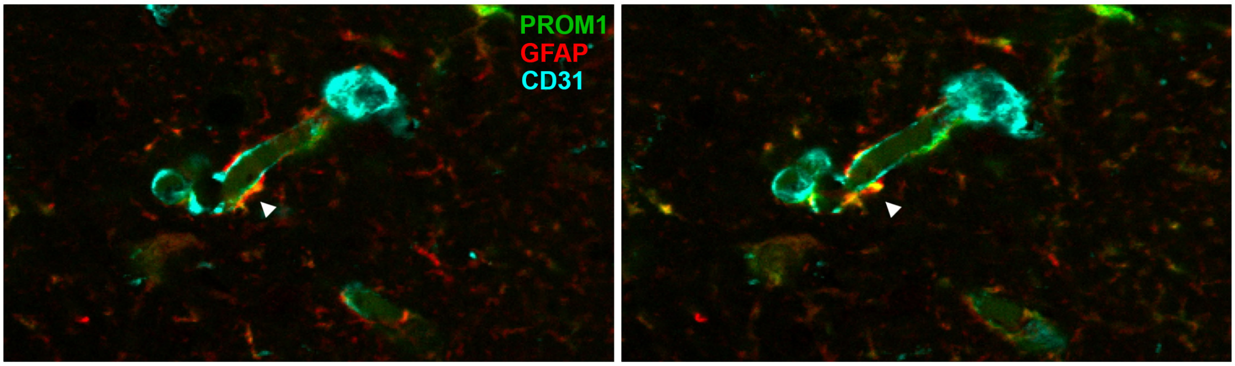

Supplementary Figure 24. GFAP+ and PROM1+ processes contact vessels in the 6-year-old human brain; related to Figure 10c and Supplementary Movie 3.
Still frames from Supplementary Movie 3 of 6-year-old human SVZ stained for GFAP, PROM1 and CD31 show a vessel with an overlapping GFAP+PROM1+ process highlighted by arrowheads.

Supplementary Table 1. Summary of type B cell markers in human versus rodent from scRNAseq and immunostaining data.

Gene or marker	Human type B markers not previously reported	Human type B (scRNAseq)	Human type B (IHC)	Human NSC (scRNAseq)	Rodent type B (scRNAseq)
S100A6	*	+	#	+	+[2, 5-7]
(LeX)	*		#		#[8]
PROM1		+	#, #[9]		
GFAP		+	#, #[10]		+[2, 7, 11]
NGFR		+	#[10]		
HES1	*	+			
NRXN3	*	+			
TIAM2	*	+			
APOE	*	+			+[2, 5-7, 11]
NTRK2	*	+			+[2, 6, 7, 11]
MT3	*	+		+	+[2, 6, 7, 11]
MALAT1	*	+		+	+[7]
BTG1	*	+		+	+[7]
CEBPD	*	+		+	+[2]
SLC1A3	*	+		+	+[7]

Red color denotes the current study, while black is previously published, * indicates a novel human type B cell marker, + indicates marker confirmation via scRNAseq or other RNA based method, and # indicates marker confirmation via immunohistochemistry (IHC) of human brain tissue. LeX is a carbohydrate epitope and not a gene, so is in parentheses. Since the LeX epitope is generated by multiple enzymes, scRNAseq analysis could not assess LeX marker expression.

References

1. Li, Z. *et al.* Transcriptional priming as a conserved mechanism of lineage diversification in the developing mouse and human neocortex. *Sci Adv* **6** (2020).
2. Cebrian-Silla, A. *et al.* Single-cell analysis of the ventricular-subventricular zone reveals signatures of dorsal and ventral adult neurogenesis. *Elife* **10** (2021).
3. Nowakowski, T.J. *et al.* Spatiotemporal gene expression trajectories reveal developmental hierarchies of the human cortex. *Science* **358**, 1318-1323 (2017).
4. Baig, S. *et al.* Identity and nature of neural stem cells in the adult human subventricular zone. *iScience* **27**, 109342 (2024).
5. Basak, O. *et al.* Troy+ brain stem cells cycle through quiescence and regulate their number by sensing niche occupancy. *Proc Natl Acad Sci U S A* **115**, E610-E619 (2018).
6. Zywitza, V., Misios, A., Bunatyan, L., Willnow, T.E. & Rajewsky, N. Single-Cell Transcriptomics Characterizes Cell Types in the Subventricular Zone and Uncovers Molecular Defects Impairing Adult Neurogenesis. *Cell Rep* **25**, 2457-2469.e2458 (2018).
7. Borrett, M.J. *et al.* Single-Cell Profiling Shows Murine Forebrain Neural Stem Cells Reacquire a Developmental State when Activated for Adult Neurogenesis. *Cell Rep* **32**, 108022 (2020).
8. Shen, Q. *et al.* Adult SVZ stem cells lie in a vascular niche: a quantitative analysis of niche cell-cell interactions. *Cell Stem Cell* **3**, 289-300 (2008).
9. Holmberg Olausson, K. *et al.* Prominin-1 (CD133) defines both stem and non-stem cell populations in CNS development and gliomas. *PLoS One* **9**, e106694 (2014).
10. van Strien, M.E. *et al.* Isolation of neural progenitor cells from the human adult subventricular zone based on expression of the cell surface marker CD271. *Stem Cells Transl Med* **3**, 470-480 (2014).
11. Codega, P. *et al.* Prospective identification and purification of quiescent adult neural stem cells from their in vivo niche. *Neuron* **82**, 545-559 (2014).
