# Supplementary material for: Human endothelial cells promote a human neural stem cell type B phenotype via Notch signaling
Source: Nat Commun. 2025 May 30;16:5031. doi: 10.1038/s41467-025-60194-6 (PMC12125299; doi:10.1038/s41467-025-60194-6)
Supplement: Supplementary file 2 — Description of Addtional Supplementary Files [file 41467_2025_60194_MOESM2_ESM.docx]

**Supplementary Movie 1.** **GFAP+ processes contact vessels in the human SVZ; related to Figure 10a and Supplementary Figure 22.** Video showing Z-stack of 18 confocal slices, each 1 µm thick, through 15-year-old human SVZ stained for GFAP and CD31.

**Supplementary Movie 2.** **GFAP+ and PROM1+ processes wrap around vasculature in the 6-year-old human brain; related to Figure 10c and Supplementary Figure 23.** Video showing Z-stack of 6 confocal slices, each 1 µm thick, through 6-year-old human SVZ stained for GFAP, PROM1 and CD31.

**Supplementary Movie 3.** **GFAP+ and PROM1+ processes contact vessels in the 6-year-old human brain; related to Figure 10c and Supplementary Figure 24.** Video showing Z-stack of 15 confocal slices, each 1 µm thick, through 6-year-old human SVZ stained for GFAP, PROM1 and CD31.

**Supplementary Data 1.** **Genes expressed by hNSPC and hEC clusters from merged media control, hEC CM, and co-culture datasets.** Genes expressed by endothelial cells, proliferating endothelial cells, neural stem cells, transitional cells, astrocyte progenitors and proliferating progenitors. Statistics were conducted in Seurat using Wilcoxon Rank Sum test with Bonferroni correction.

**Supplementary Data 2.** **Genes expressed by hNSPC clusters identified in subset analysis.** Genes expressed by neural stem cells, transitional cells, neural stem cells (Type B), astrocyte progenitors, proliferating progenitors, and early neuron progenitors. Statistics were conducted in Seurat using Wilcoxon Rank Sum test with Bonferroni correction.

**Supplementary Data 3.** **Differentially expressed genes in mouse type B cells and mouse radial glial cells.** Genes differentially expressed by mouse type B cells (mTypeB) from the Cebrian-Silla et al. study compared to mouse radial glial cells (mRGCs) from the Li et al. study. Genes with higher expression in mTypeB are in purple cells and genes with higher expression in mRGCs are in blue cells. Pct.1 is the percentage of mTypeB cells and Pct.2 is the percentage of mRGCs in which the gene is detected. Purple bolded genes are among the top 50 upregulated genes in our human type B NSPCs based on average log2 fold change. Statistics were conducted in Seurat using Wilcoxon Rank Sum test with Bonferroni correction.

**Supplementary Data 4.** **Differentially expressed genes in human type B cells and human radial glial cells.** Genes differentially expressed by human type B cells (hTypeB_a) from the Baig et al. study compared to human radial glial cells (hRGCs) from the Nowakowski et al. study. Genes with higher expression in hTypeB_a are in purple cells and genes with higher expression in hRGCs are in blue cells. Pct.1 is the percentage of hTypeB_a cells and Pct.2 is the percentage of hRGCs in which the gene is detected. A small number of mitochondrial genes were omitted due to mismatch in naming convention. Statistics were conducted in Seurat using Wilcoxon Rank Sum test with Bonferroni correction.

**Supplementary Data 5.** **Genes expressed by Type B hNSPC clusters.** Subset analysis of just type B hNSPCs was conducted, revealing two type B sub-clusters: TypeB_S1 and TypeB_S2. Genes expressed by each cluster are shown. Statistics were conducted in Seurat using Wilcoxon Rank Sum test with Bonferroni correction.

**Supplementary Data 6.** **Differential gene expression of type B hNSPCs in co-culture versus media control.** Genes upregulated (green) and downregulated (red) in type B hNSPCs in co-culture compared to media control. Statistics were conducted in Seurat using Wilcoxon Rank Sum test with Bonferroni correction.

**Supplementary Data 7.** **Enrichr analysis of type B hNSPC upregulated genes after co-culture.** Gene enrichment analysis of the top 50 upregulated genes (adjusted p-value) in type B hNSPCs in co-culture versus media control was conducted using Enrichr Gene Ontology cellular components and Panther metabolic and cell signaling pathway databases. Statistics were conducted by Enrichr using Fisher’s exact test with Benjamini-Hochberg correction.
