# Supplementary material for: Human endothelial cells promote a human neural stem cell type B phenotype via Notch signaling
Source: Nat Commun. 2025 May 30;16:5031. doi: 10.1038/s41467-025-60194-6 (PMC12125299; doi:10.1038/s41467-025-60194-6)
Supplement: Supplementary file 13 — Reporting Summary [file 41467_2025_60194_MOESM13_ESM.pdf]

nature portfolio

Corresponding author(s): B. Gutierrez and L.A. Flanagan

Last updated by author(s): 04/24/2025

Reporting Summary

Nature Portfolio wishes to improve the reproducibility of the work that we publish. This form provides structure for consistency and transparency in reporting. For further information on Nature Portfolio policies, see our Editorial Policies and the Editorial Policy Checklist.

Statistics

For all statistical analyses, confirm that the following items are present in the figure legend, table legend, main text, or Methods section.

n/a	Confirmed	
☐	☒	The exact sample size (*n*) for each experimental group/condition, given as a discrete number and unit of measurement
☐	☒	A statement on whether measurements were taken from distinct samples or whether the same sample was measured repeatedly
☐	☒	The statistical test(s) used AND whether they are one- or two-sided<br>*Only common tests should be described solely by name; describe more complex techniques in the Methods section.*
☒	☐	A description of all covariates tested
☐	☒	A description of any assumptions or corrections, such as tests of normality and adjustment for multiple comparisons
☐	☒	A full description of the statistical parameters including central tendency (e.g. means) or other basic estimates (e.g. regression coefficient) AND variation (e.g. standard deviation) or associated estimates of uncertainty (e.g. confidence intervals)
☐	☒	For null hypothesis testing, the test statistic (e.g. *F*, *t*, *r*) with confidence intervals, effect sizes, degrees of freedom and *P* value noted<br>*Give P values as exact values whenever suitable.*
☒	☐	For Bayesian analysis, information on the choice of priors and Markov chain Monte Carlo settings
☒	☐	For hierarchical and complex designs, identification of the appropriate level for tests and full reporting of outcomes
☒	☐	Estimates of effect sizes (e.g. Cohen's *d*, Pearson's *r*), indicating how they were calculated

Our web collection on statistics for biologists contains articles on many of the points above.

Software and code

Policy information about availability of computer code

Data collection	NIS Elements AR 4.51 (Nikon), Fluoview FV31s (Olympus), Keyence BZ-X8100 Viewer (Keyence), 10x Genomics v3.1 chemistry, NovaSeq 6000 (Illumina), QuantStudio7 RT-PCR (Thermo Fisher Scientific)
Data analysis	Data were analyzed using: ImageJ (NIH), NIS Elements AR 4.51 (Nikon), FastQC, 10x Genomics Cell Ranger Count v6.0.1 (alignment to human reference genome GRCh38 2020-A), Seurat package version 4.0.4 and 5.1.0, R version 4.1.0 and 4.4.2, Web-based Enrichr (Gene Ontology and Panther metabolic and cell signaling pathway databases), GraphPad Prism version 9.3.1. Code available at https://github.com/bgutie1/Nature-Communications-2025.git

For manuscripts utilizing custom algorithms or software that are central to the research but not yet described in published literature, software must be made available to editors and reviewers. We strongly encourage code deposition in a community repository (e.g. GitHub). See the Nature Portfolio guidelines for submitting code & software for further information.

Data

Policy information about availability of data

All manuscripts must include a data availability statement. This statement should provide the following information, where applicable:

- Accession codes, unique identifiers, or web links for publicly available datasets
- A description of any restrictions on data availability
- For clinical datasets or third party data, please ensure that the statement adheres to our policy

The single-cell RNA sequencing dataset generated in this study has been deposited in the NCBI's Gene Expression Omnibus (GEO) database under accession (number available after manuscript acceptance). Source data are provided with the paper.

Publicly available data utilized:
Mouse radial glial cell clusters (mRGC1, mRGC2, mRGC3) GSE143949 (Li et al., Sci Adv 2022).
Type B cell clusters (dorsal and ventral type B cell from P29 and P35 mice) GSE165554 (Cebrian-Silla A. et al., Elife 2021).
Human radial glial cell clusters (tRG, vRG, oRG, and RG-early), expression matrix and metadata files downloaded from UCSC cell browser (Nowakowski et al., Science 2017).
Human adult SVZ NSC-like cells GSE248995 (Baig et al., iScience 2024).

Research involving human participants, their data, or biological material

Policy information about studies with human participants or human data. See also policy information about sex, gender (identity/presentation), and sexual orientation and race, ethnicity and racism.

Reporting on sex and gender	Sex of cells used in the study is reported in the manuscript and in the Cell line sources section below.
Reporting on race, ethnicity, or other socially relevant groupings	*Please specify the socially constructed or socially relevant categorization variable(s) used in your manuscript and explain why they were used. Please note that such variables should not be used as proxies for other socially constructed/relevant variables (for example, race or ethnicity should not be used as a proxy for socioeconomic status).*<br>*Provide clear definitions of the relevant terms used, how they were provided (by the participants/respondents, the researchers, or third parties), and the method(s) used to classify people into the different categories (e.g. self-report, census or administrative data, social media data, etc.)*<br>*Please provide details about how you controlled for confounding variables in your analyses.*
Population characteristics	*Describe the covariate-relevant population characteristics of the human research participants (e.g. age, genotypic information, past and current diagnosis and treatment categories). If you filled out the behavioural & social sciences study design questions and have nothing to add here, write "See above."*
Recruitment	*Describe how participants were recruited. Outline any potential self-selection bias or other biases that may be present and how these are likely to impact results.*
Ethics oversight	Use of human stem cells was approved by the University of California, Irvine Human Stem Cell Research Oversight (HSCRO) committee and earlier collection of human samples for cell isolation was approved for the National Human Neural Stem Cell Resource by the Children's Hospital of Orange County Institutional Review Board with informed consent and cells available for research purposes only. All tissues and cells were acquired in compliance with NIH and institutional guidelines. Cord blood-derived human endothelial colony-forming cell-derived endothelial cells (hECs) were isolated as previously described, with informed consent and approval by UC Irvine's Institutional Review Board. Autopsy, post-mortem (less than 48 hours) gestational week 36, 2-year-old, 6-year-old, and 15-year-old human anterior horn SVZ tissue used as neuropathological controls was obtained at University of California, San Francisco (UCSF) following institutional guidelines with previous patient consent and strict observance of the legal and institutional ethical regulations set by the UCSF Committee on Human Research. Protocols were approved by the Human Gamete, Embryo and Stem Cell Research Committee (Institutional Review Board GESCR# 10-02693) at UCSF.

Note that full information on the approval of the study protocol must also be provided in the manuscript.

Field-specific reporting

Please select the one below that is the best fit for your research. If you are not sure, read the appropriate sections before making your selection.

☒ Life sciences ☐ Behavioural & social sciences ☐ Ecological, evolutionary & environmental sciences

For a reference copy of the document with all sections, see nature.com/documents/nr-reporting-summary-flat.pdf

Life sciences study design

All studies must disclose on these points even when the disclosure is negative.

Sample size	No statistical methods were used to predetermine sample sizes. Sample sizes were chosen based on data from pilot experiments.

Data exclusions	No data were excluded from analysis.
Replication	Experiments were conducted with at least three biologically independent replicates, unless stated otherwise. Exact numbers of biological experiments, technical replicates and samples are indicated in the figures and figure legends.
Randomization	Cells were randomly assigned to groups (control, conditioned media, or co-culture). Regions for quantification of cells on coverslips were randomly chosen.
Blinding	Investigators were not blinded to group allocation during data collection and analysis since co-cultures were visually distinct from monocultures and experiments were performed by a single investigator to minimize variability. For scRNA-seq analysis, data acquisition and analysis were unbiased and without prior knowledge of results.

Reporting for specific materials, systems and methods

We require information from authors about some types of materials, experimental systems and methods used in many studies. Here, indicate whether each material, system or method listed is relevant to your study. If you are not sure if a list item applies to your research, read the appropriate section before selecting a response.

Materials & experimental systems

n/a	Involved in the study
☐	☒ Antibodies
☐	☒ Eukaryotic cell lines
☒	☐ Palaeontology and archaeology
☒	☐ Animals and other organisms
☒	☐ Clinical data
☒	☐ Dual use research of concern
☒	☐ Plants

Methods

n/a	Involved in the study
☒	☐ ChIP-seq
☒	☐ Flow cytometry
☒	☐ MRI-based neuroimaging

Antibodies

Antibodies used

All antibodies are sourced commercially and described in the Experimental Procedures section.

Rabbit polyclonal IgG anti-GFAP (Agilent DAKO Cat# Z033429-2)
Goat polyclonal IgG anti-GFAP (abcam Cat# ab53554)
Mouse monoclonal IgG anti-GFAP (Sigma Aldrich Cat# G3893)
Goat polyclonal IgG anti-SOX2 (R&D Systems Cat# AF2018)
Mouse monoclonal IgG anti-CD31 (Agilent Dako Cat# M0823, Clone JC70A)
Mouse monoclonal IgM anti-CD15 (BD Biosciences Cat# 560828, Clone HI98)
Rabbit monoclonal IgM anti-S100A6 (abcam Cat# ab181975)
Rabbit polyclonal IgG anti-Aquaporin 4 (Sigma Aldrich Cat# AB3594)
Mouse monoclonal IgG anti-Map2 (Sigma Aldrich Cat# M9942)
Goat monoclonal IgG anti-DCX (Santa Cruz Biotechnology Cat# SC-80660)
Rabbit monoclonal IgG anti-Ki67 (Leica Biosystems Cat# NCL-Ki67p)
Rabbit polyclonal IgG anti-cleaved caspase (Cell Signaling Technologies Cat#9661
Alexa Fluor 488 Donkey anti-rabbit IgG (Jackson ImmunoResearch Cat# 711-545-152)
Alexa Fluor 594 Donkey anti-mouse IgG (Jackson ImmunoResearch Cat# 715-585-151)
Alexa Fluor 633 Donkey anti-goat IgG (Thermo Scientific Cat# A-21082)
AlexaFluor 647 Donkey anti-mouse IgM (Jackson ImmunoResearch Cat# 715-605-020)
AlexaFluor 594 Donkey anti-goat IgG (Jackson ImmunoResearch Cat# 705-545-147)
AlexaFluor 488 donkey anti-mouse IgG (Jackson ImmunoResearch Cat# 715-585-151)

Validation

All antibodies were optimized and validated commercially. Information on validation can be found on the supplier's website. In all cases, antibody staining matched the expected subcellular localization of the antigen.
GFAP (Z0334) https://www.agilent.com/store/en_US/Prod-Z033429-2/Z033429-2
GFAP (ab53554) https://www.abcam.com/products/primary-antibodies/gfap-antibody-ab53554.html
GFAP (G3893) https://www.sigmaaldrich.com/US/en/product/sigma/g3893
SOX2 (AF2018) https://www.rndsystems.com/products/human-mouse-rat-sox2-antibody_af2018
CD31 (M0823) https://www.agilent.com/en/product/immunohistochemistry/antibodies-controls/primary-antibodies/cd31-endothelial-cell-(concentrate)-76539
CD15 (560828) https://www.fishersci.com/shop/products/anti-cd15-percp-cy-5-5-clone-hi98-bd-50-tests-percp-cy5-5/bdb560828
S100A6 (ab181975) https://www.abcam.com/products/primary-antibodies/s100-alpha-6pra-antibody-epr13084-69-ab181975.html
Aquaporin 4 (AB3594) https://www.sigmaaldrich.com/US/en/product/mm/ab3594
Map2 (M9942) https://www.sigmaaldrich.com/US/en/product/sigma/m9942
DCX (SC-80660) https://www.scbt.com/p/doublecortin-antibody-c-18
Ki67 (NCL-Ki67p) https://shop.leicabiosystems.com/us/ihc-ish/ihc-primary-antibodies/pid-ki67
Cleaved Caspase-3 (https://www.cellsignal.com/products/9661/datasheet?images=1&protocol=0&srsltid=AfmBOoqm3WpLY0Q1pH-OZ4VM0-9XsPkG4zZF6Dpnlm1f937K3-FBB0wN)
Alexa Fluor 488 (711-545-152) https://www.jacksonimmuno.com/catalog/products/711-545-152

Alexa Fluor 594 (715-585-151) https://www.jacksonimmuno.com/catalog/products/715-585-151
Alexa Fluor 633 (A-21082) https://www.thermofisher.com/antibody/product/Donkey-anti-Goat-IgG-H-L-Cross-Adsorbed-Secondary-Antibody-Polyclonal/A-21082
AlexaFluor 647 (715-605-020) https://www.jacksonimmuno.com/catalog/products/715-605-020
AlexaFluor 594 (705-545-147) https://www.jacksonimmuno.com/catalog/products/705-585-147
AlexaFluor 488 (715-585-151) https://www.jacksonimmuno.com/catalog/products/715-585-151

Eukaryotic cell lines

Policy information about cell lines and Sex and Gender in Research

Cell line source(s)	Human fetal brain-derived neural stem/progenitor cells were isolated from cadaveric brain cortices by the National Human Neural Stem Cell Resource and designated as SC27 (male, 23 weeks gestation), SC30 (male, 25 weeks gestation), and SC23 (male, 23 weeks gestation) (Schwartz, P.H. et al., 2003), human endothelial colony-forming cell-derived endothelial cells (female) were isolated from cord blood with the approval of University of California Irvine's Institutional Review Board (Melero-Martin, J.M. et al., 2015), human brain microvascular endothelial cells (hBMECs) were commercially obtained (lot# 31155, male, Cat# 1001, ScienCell, Carlsbad, CA, USA), and normal human lung fibroblasts (female, Cat# CC-2512, Lot 20TL329334, Lonza, Morrisville, NC, USA) were commercially obtained.
Authentication	All cells have been authenticated by original sources and by in-house morphological observation, immunocytochemistry, and in-vitro differentiation, which are carried out routinely to monitor cell phenotype. Further cell validation was provided by matching of scRNAseq expression patterns to published datasets of similar cells.
Mycoplasma contamination	Mycoplasma contamination is routinely tested with PCR-based mycoplasma detection kit (Universal Mycoplasma Detection kit, ATCC, Cat: 30-1012K). All cells were negative for mycoplasma.
Commonly misidentified lines (See ICLAC register)	No commonly misidentified cell lines were used.

Plants

Seed stocks	*Report on the source of all seed stocks or other plant material used. If applicable, state the seed stock centre and catalogue number. If plant specimens were collected from the field, describe the collection location, date and sampling procedures.*
Novel plant genotypes	*Describe the methods by which all novel plant genotypes were produced. This includes those generated by transgenic approaches, gene editing, chemical/radiation-based mutagenesis and hybridization. For transgenic lines, describe the transformation method, the number of independent lines analyzed and the generation upon which experiments were performed. For gene-edited lines, describe the editor used, the endogenous sequence targeted for editing, the targeting guide RNA sequence (if applicable) and how the editor was applied.*
Authentication	*Describe any authentication procedures for each seed stock used or novel genotype generated. Describe any experiments used to assess the effect of a mutation and, where applicable, how potential secondary effects (e.g. second site T-DNA insertions, mosiacism, off-target gene editing) were examined.*
